# Hydraulic flow unit and rock types of the Asmari Formation, an application of flow zone index and fuzzy C-means clustering methods

**DOI:** 10.1038/s41598-024-55741-y

**Published:** 2024-02-29

**Authors:** Seyedeh Hajar Eftekhari, Mahmoud Memariani, Zahra Maleki, Mohsen Aleali, Pooria Kianoush

**Affiliations:** 1 Department of Earth Sciences, Science and Research Branch, Islamic Azad University, 1477893855 Tehran, Iran; 2 Department of Petroleum and Mining Engineering, South Tehran Branch, Islamic Azad University, Tehran, Iran; 3 National Iranian Oil Company, Exploration Directorate (NIOC-EXP), Tehran, Iran

**Keywords:** Asmari reservoir, Mansouri field, Hydraulic flow units, Rock types, Flow zone index, Fuzzy c-means, Solid Earth sciences, Geology, Petrology, Sedimentology

## Abstract

*Rock types* are the reservoir's most essential properties for special facies modeling in a defined range of porosity and permeability. This study used clustering techniques to identify rock types in 280 core samples from one of the wells drilled in the Asmari reservoir in the Mansouri field, SW Iran. Four hydraulic flow units (HFUs) were determined for studied data utilizing histogram analysis, normal probability analysis, and the sum of squared errors (SSE) statistical methods. Then, two flow zone index (FZI) and fuzzy c-means (FCM) clustering methods were used to determine the rock types in the given well according to the results obtained from the HFU continuity index acts in-depth. The FCM method, with a continuity number of 3.12, compared to the FZI, with a continuity number of 2.77, shows more continuity in depth. The relationship between permeability and porosity improved considerably by utilizing HFU techniques. This improvement is achieved using the FZI method study. Generally, all samples increased from 0.55 to 0.81 in the first HFU and finally to 0.94 in the fourth HFU. Similar flow properties in an HFU characterized the samples. In comparison, the correlation coefficients obtained in the FCM method are less than those in the general case of all HFUs. This study aims to determine the flowing fluid in the porous medium of the Asmari reservoir employing the c-mean fuzzy logic. Also, by determining the facies of the rock units, especially the siliceous-clastic facies and log data in the Asmari Formation, the third and fourth flow units have the highest reservoir quality and permeability. Results can be compared to determining HFU in nearby wellbores without cores.

## Introduction

A *flow unit* controls the fluid flow as a separated zone with sideward continuity between wellbores and internal reservoirs' consistent factors. Furthermore, the term "*rock type classification*" was first coined by Archie^1^ and later used by many scholars and engineers. Archie first defined the classification of rock types as units of rock formed under the same sedimentary conditions^2–5^. Hydraulic flow units (HFUs) are sideward continuity of the reservoir units with invariant geological properties containing fluid flow behavior in pore media^2,3^. Amaefule et al.^2^ defined the concept of an HFU as a method for estimating permeability in reservoir and non-reservoir zones. Gomes et al.^4^ emphasized the importance of the main facies, sedimentary environments, the process of later diagenesis, and the relationship between rock and fluid by specific core analysis (SCAL) to establish relationships between geological facies, petrophysical groups, and rock classification^5–7^. Kharrat et al.^8^ used artificial neural networks (ANNs) and geostatistical data to model HFUs to estimate permeability and rock classification. Hollis et al.^9^ used the specification of the cavity system in heterogeneous carbonates as an alternative strategy to the well-known methods used to determine rock groups. Permadi et al.^10^ conducted experiments on two models of carbonate and sandstone with different wettability. Chandra et al.^11^ effectively integrated reservoir rock clusters and simulations using well drilling. Using rock bands determination and well drilling, the X field reservoir simulation model was improved, and therefore, the accuracy of in situ fluid computation was also improved^12–14^. Ghadami et al.^15^ Studied Trojan-porosity modeling, the determination of reservoir rock groups, and the unification of hydraulic flow in a large carbonate reservoir. In 2016, rock groups of a rigid gas sandstone were determined in the Lance Formations and the Massif from the Yunus field^15^.

Mirzaei-Paiaman and Saboorian-Jooybari^16^ proposed a spontaneous adsorption-based method for characterizing pore structure and its application in pre-SCAL sample selection and rock group determination. They used the flow zone index (FZI) for spontaneous absorption (COUCSI). Moradi et al.^17^ identified rock groups using geological and petrophysical data in the Asmari reservoir in the Aghajari oilfield, SW Iran. Using data mining techniques, Gonçalves et al.^18^ predicted carbonate rock groups from NMR responses. Their experiments show that combining pre-processed strategies with classification algorithms can increase prediction accuracy to 97.4%.

Mahjour et al.^19^ used three methods of Testerman statistical zonation, FZI, and cluster analysis to pinpoint flow units and estimate average porosity and permeability in the Tabnaak gas field in southern Iran. Yasmaniar et al.^20^ utilized ANN to determine the permeability of different rock types, employing the HFU concept^21^. Oliveira et al.^22^ demonstrated that an inter-clustering procedure is advised when selecting data points associated with representative volumes and local spots characterizing HFUs. In 2020, rock type and HFUs were used as successful tools for reservoir characterization of the Bentiu-Abu Gabra sequence, Muglad basin, SW Sudan^23,24^. Machine learning is effectively used by Man et al.^25^ to boost the prediction of permeability and reduce uncertainty in reservoir modeling. Recently, various established techniques and machine learning algorithms were examined in specifying HFUs, and the implementation of each method was assessed^21,26–29^. Salavati et al.^30^ used HFUs, multi-resolution graph-based clustering (MRGC), and fuzzy c-mean (FCM) clustering methods to specify rock types. Al-Ismael and Awotunde^31^ used differential evolution optimization and two-stage clustering approaches to identify HFUs to maintain a crucial process in reservoir characterization. Omeje et al.^32^ applied an FZI to discriminate an aquifer in west Nigeria into HFUs. Djebbas et al.^33^ applied the adaptive-neuro-fuzzy-inference system (ANFIS) algorithm to calculate FZI in multivariate nonlinear datasets in the Sif Fatima oilfield, Algeria. Mohammadinia et al.^26^ used different intelligent methods to determine the HFUs of the Kazhdumi Formation in SW Iran. Instead of utilizing only two parameters of permeability and porosity, further data acquired from wireline logging are employed. Eventually, a novel approach has been made for estimating pore size dispersion and capillary pressure in the hydrocarbon zone through an HFU framework utilizing an NMR log^34^. The workflow establishes a powerful and worthwhile procedure for NMR T2 distribution correction in the hydrocarbon zone for uncored and partially cored wells^35^.

The FCM clustering is based on minimizing an objective function describing the space from any considered data point to a cluster center weighted utilizing that data point's membership valuation. This algorithm repositions objects between clusters until the objective function cannot be reduced additionally. The FCM approach utilizes a fuzzy membership that allocates a membership degree for every class^36–38^. It has rarely been used in HFUs, but the most critical literature reviews have been presented in recent years. A broad survey on FCM and its utilizations was presented by Nayak et al.^39^ for its application in over a decade. Mohebian et al.^40^ applied Nero-fuzzy to predict FZI, fuzzy logic, and neural network to employ well-derived FZI logs from some wells to assess intelligent models in the Surmeh reservoir. Mosavi et al.^41^ propose an FCM to divide ungauged and gauged watersheds into homogenous classes depending on various climatic and topographical factors. Duy Thong et al.^42^ presented a technique of employing FCM clustering to categorize the core data and well logs in clusters and then perform inversion for each cluster. Mausor et al.^43^ proposed a key for a missing value as data imputation.

Recently, other clustering techniques like K-means and hierarchical algorithms were implemented by Di Nunno et al.^44^ and Di Nunno and Granata^45^ to determine precipitation and evapotranspiration in some homogenous regions. Furthermore, the Gaussian mixture model (GMM) clustering algorithm was employed by Najafi-Silab et al.^46^ to estimate rock types in a sandstone and carbonate reservoir. Hierarchical clustering was performed based on profile validation and geological information as a robust and practical approach to data clustering. Furthermore, MRGC was employed to automatically define the optimal number of clusters, and the level of electrical facies clusters could be specified based on actual requirements. This approach makes the MRGC method more robust than other hierarchical clustering algorithms^22,47^.

Moreover, as per the recent literature review, Hossain et al.^48^ utilized FCM in subsurface electrofacies lithological classification. Hussain et al.^49^ identified rock types for lithofacies prediction by machine learning. Xing et al.^50^ employed machine learning of core and log data for rock-type classification. Krivoshchekov et al.^51^ characterized complex carbonate reservoirs utilizing rock types. Eventually, Kumar et al.^52^ employed FCM clustering as an unsupervised machine learning algorithm for a multi-scale geological mapping of potential field data under the sediment litho-units^52,53^. In previous literature review studies, each of the K-means, FZI, FCM, and HFU methods was employed separately or in combination with other clustering methods, such as ANN and Hierarchical in sandstone and limestone formations. In this study, the integration of K-means and FZI methods as deterministic clustering and FCM as fuzzy clustering was done to determine hydraulic units. Other clustering methods are also suggested for future studies.

*Mansouri field* is a strategic reservoir supplying hydrocarbon resources from sandstone and limestone zones. This research was carried out in the first exploratory well in the west of Mansouri field, where a combination of the FZI and FCM as fuzzy and deterministic methods was used more broadly and completely by comparing their centroid and median using the data of the drilling core in a wide range of the Asmari Formation. In the exploratory well located in the west of Mansouri field, after passing through the Gachsaran Formation due to the severe drilling fluid losses in the upper portions of the Asmari Formation, it was not possible to core drilling, and the rock type and hydraulic flow unit model was done employing drilling cuttings and logs.

This study aims to specify the reservoir modeling and sedimentary environment for the Asmari reservoir in the Mansouri oilfield, utilizing techniques to identify rock typing, flow units, and electrofacies. It has been carried out in two stages. This manuscript is the result of the first part of the studies. In this study, core sample plugs are prepared from 280 core samples of the Asmari Formation, and their porosity and permeability are determined by measuring devices. Using MATLAB R2021a software, the FZI logarithmic data and histogram analysis are performed for each sample, and the number of HFUs is determined based on the normal distributions. The sum of squared errors (SSE) parameter is employed to reduce the heightened likelihood of error in computing the number of HFUs. This method performs K-means cluster analysis for the data employing MATLAB software, first assuming the number of flow units equal to 1 (HFU = 1), then linear regression analysis is performed on the data and calculates the SSE. The optimal number of HFUs in the FZI method is determined by plotting the HFU number against SSE.

In the FCM clustering process, each rock group will have its statistical characteristics and amplitude of its porosity and permeability variations, separating it from other groups. In addition, in porosity versus permeability cross plots, each group is well separated from the other groups, and there is no overlap. In this case, integrating the FCM and FZI, each rock type represents a facies with a specific range of porosity and permeability. Also, a zoning methodology for the studied exploratory well west of the study field is considered. The results show that the FCM method did not enhance the relationship between the petrophysical parameters of the reservoir in all HFUs and caused a decrease in the relationship between porosity and permeability.

## Geological setting

### Location and structural geology of study field

**Figure 1:**
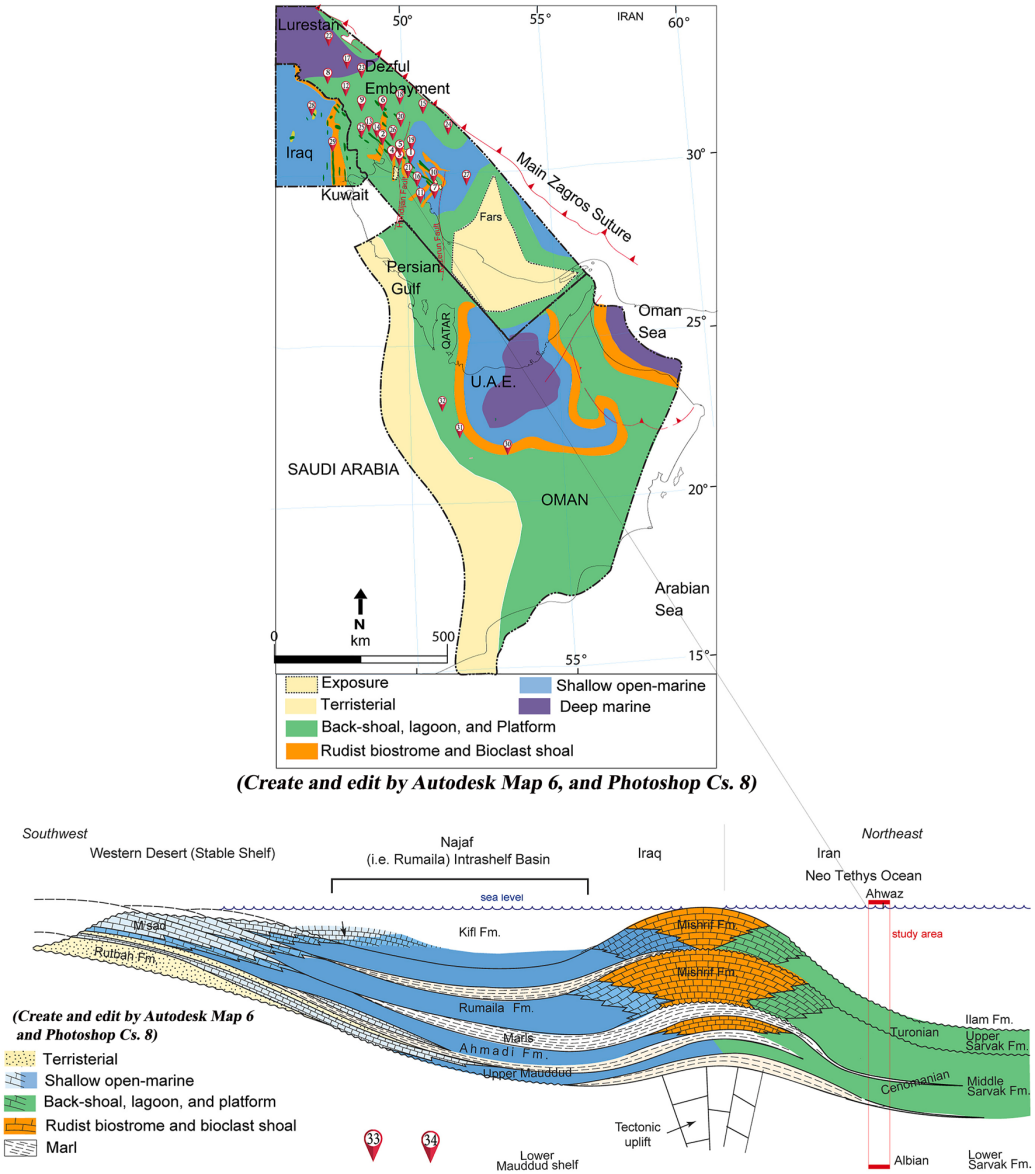
The suggested depositional environment of the Zagros and contiguous basins in the mid-Cretaceous^63^.

**Figure 2:**
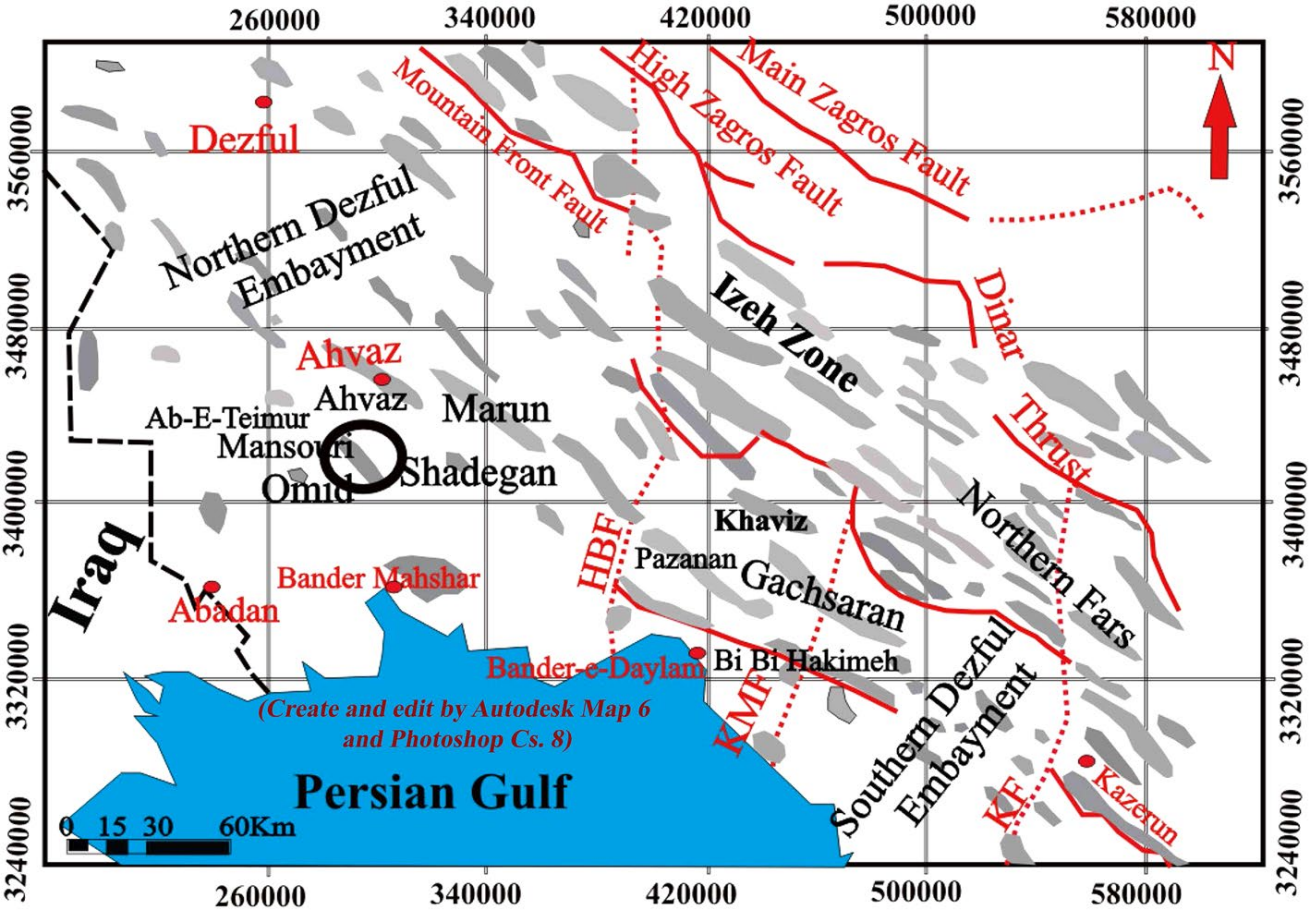
The Mansouri oilfield and adjacent fields^26,30^.

Calcareous deposits of the middle-late Cretaceous (the Santonian, Turonian, Cenomanian, and upper Albian stages), comprehended as the Bangestan reservoir (Ilam and Sarvak Formations), are amongst the considerable oilfields in the Zagros Basin, which contain a sizeable prolific hydrocarbon reservoir. Mansouri field in the southernmost part of the north Dezful zone, about 45 km south of Ahwaz, is located approximately on the border of the Arabian plate, and quaternary alluviums represent the Zagros plate and its surface outcrop (Fig. 1). Mansouri field is encountered in the north of the Ahwaz field, in the west, in the vicinity of the Abteymur and Susangerd fields, and in the northeast of the Shadegan field. The axial trend of this field is from the northwest to the southeast (the general Zagros trend) and lies between 48°–52° east longitude and 30°–32° north latitude (Fig. 2). Mansouri field is located in a flat zone just off the foot of the foothills and was discovered by seismic exploration in 1963. Based on the seismic and structural maps of the Mansouri field, it is an anticline with gentle and low slopes in the northwest–southeast (NW–SE) direction. The northern slopes are slightly higher than the southern slopes, respectively^54,55^.

Furthermore, 5°–6°, the slope of the eastern and western slopes is about 1°–5°. The study of geophysical maps and the information on drilled wells show no evidence of fault or disruption in the field, and it is generally mild in structure (Fig. 2). Mansouri's field in the horizons of Asmari is about 42 km long. It has a variable width of up to 6 km in the middle of the field and an average of 4.5 km, which decreases to the east and west slopes. The dimensions of the reservoir at the contact surface of water and oil (2272 m below sea level) are 30 km long and 3.5 km wide, stretching northwest–southeast^28,56–60^.

The limestone Asmari Formation is the most critical Zagros sedimentary reservoir. In terms of sedimentation, it began in the former Oligocene and continued until the former Miocene. This formation is the shallow horizon of oil producers in SW Iran and forms the most essential rock reservoir in Dezful's embayment. The Asmari Formation geologically consists of sandstone, dolomite, limestone, and low to average clay minerals. This formation is characterized by light brown, complex, and fine-grained dolomite with lime layers, followed by medium to coarse grains, often without cement or calcareous and dolomite and sandy cement. White limestone to light grey is observed in the formation's lower part. The formation's upper and lower limit has the same slope. In addition to the Asmari reservoir and sandstone section of Ahwaz, the Bangestan reservoir (Sarvak and Ilam Formations) are also present in this field.

Characteristics affecting reservoir descriptions are microfacies, sedimentary environments, tectonic conditioning, and diagenetic processes. Kiaei et al.^61^ and Kadkhodaie and Kadkhodaie^62^ explored that sedimentary environments and microfacies control the formations' porosity and mineralogy. However, the microfacies study develops researchers' understanding of the source and precedent of carbonate reservoirs; there has yet to be exhaustive research on the seismic stratigraphy of the reservoir characteristics. Furthermore, integrating the geochemical and microfacies data can enable geoscientists and petroleum engineers to comprehend reservoir characterizations satisfactorily^3,61–63^. Figure 1 shows the chronostratigraphic scheme of the sediments which is identical to the Bangestan reservoir (the Santonian to upper Albian) in the Arabian Plate and the Zagros Basin. From petrographic investigations of core data and thin sections, the Sarvak Formation consists of back shoal and shoal, flat tidal facies, lagoon, and correspondingly shallowing facies of rudist biostrome. Furthermore, the Ilam Formation illustrates open and deep marine facies. Generally, the Sarvak and Ilam Formations demonstrate evidence of an internal calcareous platform with a carbonate ramp and an interior shelf, respectively^28,54,55,64,65^.

### The Asmari Formation stratigraphy

**Figure 3:**
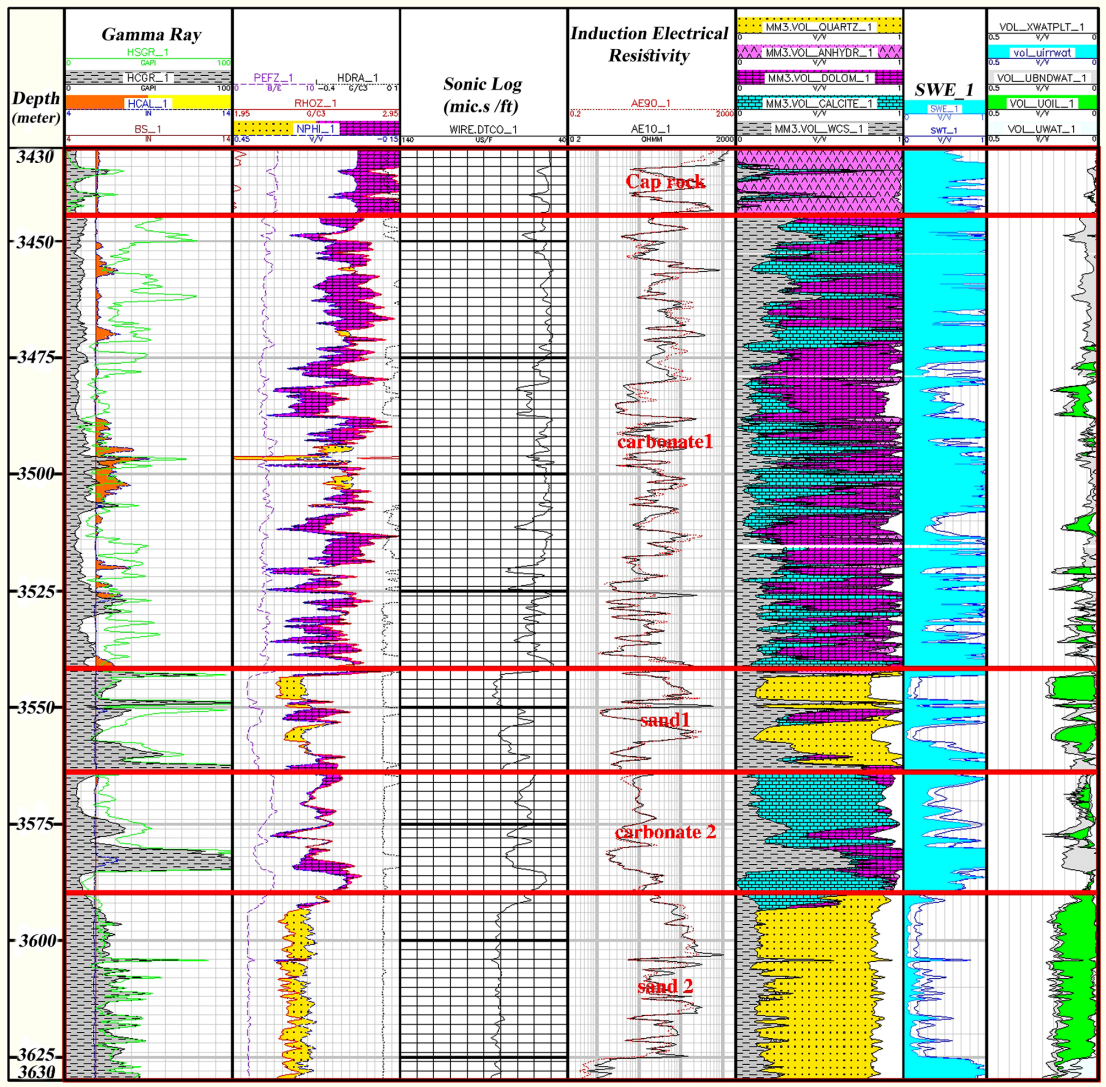
Reservoir zonation sequences of the Asmari Formation according to lithological alteration in the studied well-A employing core, cutting, and log data. Out of the total of 5 investigated zones, in zones 2 (3560–3444.5 m) and 4 (3585–35,705 m), it was not possible to coring because of the lost circulation, and only the data of the drilling cuttings, and log were used. Zone 1 includes anhydrite (pink color), dolomite (purple color), and limestone (blue color), Zone 2 includes dolomite and sandstone (yellow color), Zone 3 mainly includes sandstone, shale and limestone, Zone 4 includes limestone and Ahwaz sandstone, and finally, zone 5 contains mainly sandstone and limestone..

In the sequence of oil/gas wells, zoning is one procedure that segregates the sequence studied into zones with common conditions (geological or reservoir conditions, etc.). This section used log data to represent the Asmari Formation in the Mansouri Field accurately. The lithology was assessed and evaluated in each sequence utilizing updated and revised lithology cross-sections and logs (neutron-density, Rho-U plot, MID plot, and MN plot). Finally, using the probabilistic method, the petrophysical parameters were calculated in the whole sequence, and the average of these parameters was calculated in the whole well and each zone. The shear boundaries for the carbonate and sandstone sequences of the Asmari Formation are presented in Table 1. The cut of sandstone and carbonate values are determined from prepared well logs and calculating shale volume, saturation of water, porosity percent, and oil volume. Zones with typical reservoir geology (lithology) were studied in the well sequences using read logs (Fig. 3). The Asmari Formation has been split into five zones based on petrophysical results.

**Table 1:** Cutting limits for carbonate and sandstone sections utilizing shale volume, water saturation, porosity, and oil volume.

Parameter	Type	Cut off _(%)_ _CARBONATE_	Cut off _(%)_ _SANDSTONE_
PHIE	≥	4.5	8
SWE	≤	50	50
Vsh	≤	20	30

Zone 1 (3444.5–3427.5 m): this zone exists in all drilled wells. The central part of the zone consists of dolomite plus a thick layer of limestone. Limestones are mostly cream to light brown and cream to gray, semi-hard to hard, fine-grained, micro-crystalline with anhydrite, chert, mudstone argillic to packstone.

Zone 2 (3560.5–3444.5 m): this zone is present in all wells. The lithology of this zone mainly consists of dolomite and sandstone.

Zone 3 (3570–3560.5 m): its dominant lithology includes shale, limestone, and sandstone.

Zone 4 (3585–3570.5 m): this zone exists in all wells, and most of it contains a barrier/beach ridge and is likely to be associated with the Ahwaz sand dunes. Much of the lithology of this zone is sandstone and shale.

Zone 5 (3630–3585 m): this zone cannot be identified in all wells due to a lack of logging data. The main lithologies in this zone are shale, sandstone, and limestone.

## Methodology

**Figure 4:**
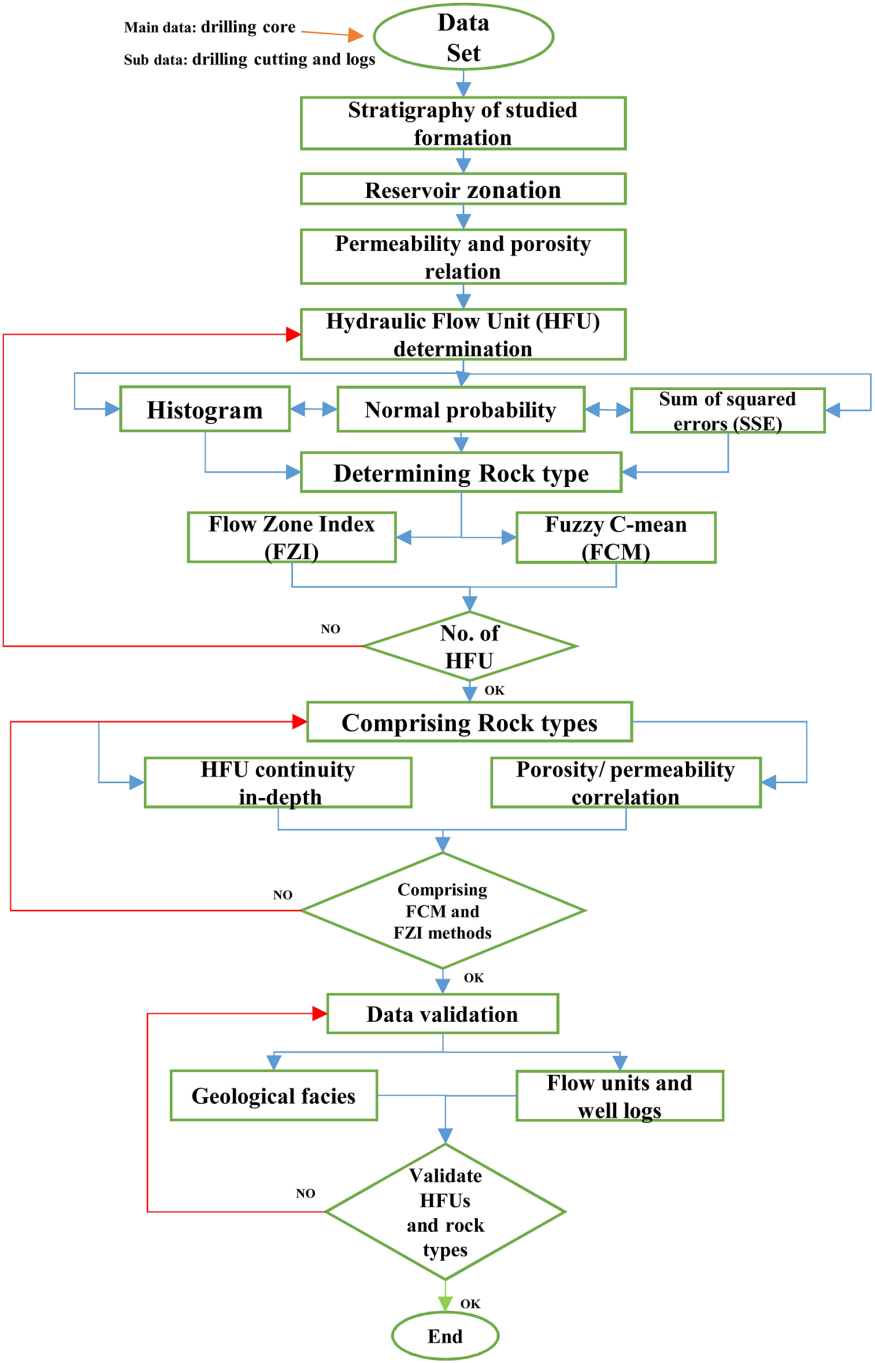
General flowchart of the study according to log and core data, rock type, and HFU methods in the studied well.

In this study, 280 core samples (acquired from one of the wells of the Mansouri oilfield) were selected to determine HFUs. Furthermore, information on permeability, porosity, and structural properties was recorded. The general flowchart of this study is presented in Fig. 4.

### Permeability and porosity relationship

Matrix permeability is related to porosity and the specific surface of rocks, as expressed by the Kozeny^66^ equation (Eq. 1):

1 $$K = \frac{{0.101\varphi^{3} S^{2} }}{{r_{i} - \varphi^{2} V}}$$

where K: matrix permeability (mD); φ: matrix porosity (%); S^2^/V: specific surface of pores in rocks (cm^2^/ cm^3^);

r_i_: dimensionless constitutional factor related to pore geometry and fluid flowing path per length in the porous medium. Kozeny 's relation is a fundamental and well-known relationship that expresses permeability as a function of the grains' porosity and specific surface area.

### Hydraulic flow units (HFUs) determination

A flow unit is a volume of reservoir rock that is continued and predictable laterally and vertically, and the geological and petrophysical characteristics affecting the fluid flow within it are constant and distinctly different from other rock volumes^8,67^. HFUs are mainly related to the distribution of geological facies. However, they do not necessarily correspond to the boundaries of these facies. Therefore, these units cannot be connected vertically. The main techniques for specifying the HFU number include histogram analysis, normal probability analysis, and the SSE. These mentioned parameters will be discussed in the following section.

#### Histogram analysis

In this method of analysis, the logarithm of HFU values ​​ will be obtained after acquiring the FZI values ​​of each sample, and then using the MATLAB R2021a [www.mathworks.com/products] software, the logarithmic data of the FZI of the histogram analysis is performed. Based on the HFUs' rules, the distribution of the logarithm of the FZI in each HFU is the normal distribution. This method applies this principle and determines the number of HFUs^63,68^.

#### Normal probability analysis

In this analysis, the logarithmic values ​​of the FZI are calculated. Based on the HFUs regulations, the normal probability logarithm of the FZI in each HFU is linearly distributed. This method uses this principle and determines the number of HFUs.

#### Sum of squared errors (SSE)

In this analysis, the working method is as follows: first, the number of categories is assumed to be 1 (HFU = 1), and the K-means cluster analysis is performed, then linear regression analysis is performed on the data and the value. Then, the SSE was calculated. This work was done the same way for the number of other categories, and finally, a graph of the SSE against the number of categories was drawn. According to the diagram, with the increase in the number of HFUs, the total amount of errors decreased. However, from one value to the next, the changes in the SSE were not noticeable and can be neglected. This value is the HFUs' optimal number.

### Methods for determination of rock types

After determining the number of HFUs, two methods are used to determine rock groups, which are discussed below.

#### Flow Zone Index (FZI) method

According to Zahaf and Tiab^53^'s findings, an HFU is continuous throughout the specific volume of the reservoir, which practically has the physical stability of rock and fluid properties. This flow unit uniquely describes the static and dynamic interactions with the well wall. Based on microscopic measurements and core samples, El-Sayed et al.^68^ developed a method to identify and describe formations with similar hydraulic properties or flow units. The rock's hydraulic quality is influenced by morphology, mineralogy, specific surface, tortuosity, radius, and pore geometry side by side with textural parameters such as shape, grain size, packing, and sorting. The same pore attribute selection samples can be clustered in a comparable hydraulic unit. The borders of rock units with unprecedented changes in flow properties can be proper for reservoir engineering^24,69,70^.

The FZI method is the most commonly used method for determining rock Types. In this method, unlike other methods, the user has no role in determining the results, and all the steps are based on mathematical models.

Kozeny^66^ has extracted one of the most essential and prominent relationships that express permeability as a system of porosity and specific surface zone. The generalized form of the Kozeny–Carman equation is as Eq. (2)^71^:

2 $$K = (\frac{1}{{\mathop f\nolimits_{g} \tau \mathop {\mathop S\nolimits_{{\mathop v\nolimits_{gr} }} }\nolimits^{2} }})\frac{{\mathop \varphi \nolimits^{3} }}{{\mathop {(1 - \varphi )}\nolimits^{2} }}$$

where K: permeability; Ø = fractional porosity; τ is the electrical tortuosity (measured from electrical resistivity); f_g_ is a shape factor, and Sv_gr_ is the grain's specific surface area.

The purpose is to avoid measuring these microscopic effects by assembling these parameters into a single variable called the flow zone index (FZI).The following parameter can introduced the flow zone and reservoir quality indexes (Eqs. 3–4):

3 $$RQI(\mu m) = 0.0314\sqrt {\frac{K}{\varphi }}$$

where RQI is the reservoir quality index (expressed in micrometers). It is an approximate index of the average hydraulic radius in the reservoir rock and is the key to the hydraulic units that correlate porosity, permeability, and capillary pressure.

Φ_z_ is the ratio of pore volume to grain volume and is defined as Eqs. (4), (5) and (6):

4 $$\mathop \varphi \nolimits_{z} = \frac{{\mathop \varphi \nolimits_{e} }}{{1 - \mathop \varphi \nolimits_{e} }}$$

FZI is considered an indicator of flow zone:

5 $$FZI(\mu m) = \frac{1}{{\sqrt {\mathop F\nolimits_{s} \mathop \tau \nolimits^{2} \mathop S\nolimits_{{\mathop v\nolimits_{gr} }}^{2} } }} = \frac{RQI}{{\mathop \varphi \nolimits_{z} }}$$

By taking the logarithm of both sides of the equation, we could write Eq. (6):

6 $$LogRQI = Log\mathop \varphi \nolimits_{z} + LogFZI$$

Equation (6) shows a straight line with the same slope on the logarithmic plot of RQI in terms of Φ_z_. The intersection point of this straight line at Φ_z_ = 1 is the FZI. Samples with different FZI values correspond to other parallel lines. Samples on a straight line have similar features and form a single flow unit. Straight lines with a slope equal to unity should initially be anticipated for sandstone formations without shale. More extensive slopes characterize shale-bearing formations. FZI is a unique parameter that includes geological properties, rock texture, and mineralogy in its geometry and facies structure.

Generally, rocks containing detrital materials have porous layering, and porous joints are filled with clays and fine graining, so they show a low FZI value. On the other hand, sands with low amounts of shale, coarse and fine graining, low specific surface area, low shape factor, and low twist degree show high FZI. Different sedimentary environments control diagenetic processes and FZI geometry. To obtain an equivalent value of FZI for each group according to Eq. (6) when plotting RQI and porosity ratio in a logarithmic graph, it must obtain a line with a constant slope of 45°, which is zero at (that is) the value. As a result, LogRQI equals LogFZI. This method can obtain FZI equivalent to each HFU^19,24,68^.

#### Fuzzy C-means (FCM) method

Clustering is a considerably crucial method of individual learning. K-means algorithm selects the initial centroids randomly where an inappropriate centroid selection issue is made. Thus, fuzzy c-mean logic is presented to access the improved distance calculation in a minimum time. In fuzzy clustering, any sample points are distributed into subgroups that cluster centers characterize. Individual data points belong to a cluster center with a degree defined by the membership grade. An FCM clustering method has been proposed to decipher the issue of allocating each data to a particular cluster in each iteration.

**Figure 5:**
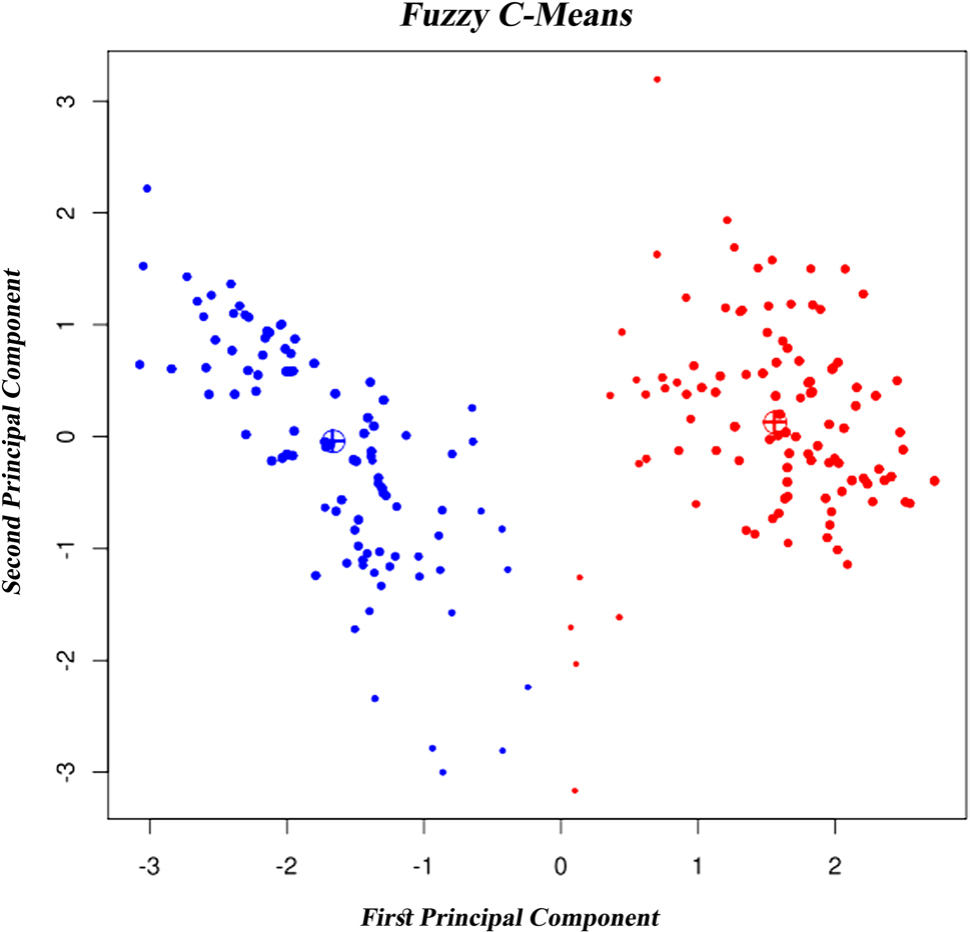
Sample of the FCM Clustering method^30,38^.

In *fuzzy clustering*, an object can be more than one cluster member. For J clusters, m_1_, m_2_, m_3_,…, and m_j_ is the possibility of an object i belonging to each cluster. These values are between 0 and 1, and their sum is 1. For this procedure, membership is distributed across all clusters. The benefit of this clustering is that each object does not have to achieve a specific cluster, and its weakness is that there is considerably more data interpretation. The cluster with a higher degree belongs to the objects close to the center of a cluster with more probability than those of the edges objects^39,43,52^. A Sample of FCM Clustering is shown in Fig. 5.

The FCM algorithm was initially presented by Bezdek^2^ to enhance premature clustering processes. FCM algorithm begins with an initial guess for the cluster centers (c_i_.), which are intended to mark the mean location of each cluster. Further, the algorithm assigns every data point a membership grade for each cluster, where u_ij_ is the degree of membership of object j (x_j_) in cluster i. Equations (7) to (9) update the membership u_ij_ and the cluster centers^39,52^. In FCM, the objective is to minimize the Eq. (7):

7 $$\mathop J\nolimits_{m} = \sum\limits_{j = 1}^{N} {\sum\limits_{i = 1}^{c} {\mathop u\nolimits_{ij}^{m} \mathop {\left\| {\mathop x\nolimits_{j} - \mathop v\nolimits_{i} } \right\|}\nolimits^{2} } }$$

where u_ij_ denotes the membership of pixel x_j_ in the j_th_ cluster, v_i_ is the i_th_ cluster center, $|| \cdot ||$ is a norm metric, and m is a constant. The parameter m handles the fuzziness of the resulting partition, and m = 2 is utilized in this study. Then, the following condition must be observed (Eq. 8):

8 $$\sum\limits_{i = 1}^{C} {u_{ij} } = 1$$

The complete procedure of this algorithm is as follows:

A) Determine the initial values for c (number of clusters), m (fuzzy value of the algorithm), and v (initial centers for each cluster).
B) Calculate the amount of belonging to each cluster.
C) Calculate the number of new centers for each cluster.

This iteration is founded on minimizing the following objective function that describes the distance from any provided data point to a cluster center weighted utilizing that data point's membership grade (Eq. 9):

9 $$\begin{gathered} Obj.Func = \sum\limits_{i = 1}^{c} {\sum\limits_{j = 1}^{n} {u_{ij}^{m} } } \left\| {\left. {x_{j} - c_{i} } \right\|} \right.^{2} \hfill \\ 1 \le i \le c \hfill \\ 1 \le j \le n \hfill \\ \end{gathered}$$

The output of the FCM method is the coordinates of the batch centers and the U matrix, where the membership functions of each point in each cluster are specified. In the FCM fuzzy clustering algorithm, the number and centers of the clusters are first determined by the user. The quality of this algorithm strongly depends on the initial number of clusters and the initial location of the cluster centers^36,72^.

### Limitations and advantages of the methods

After reviewing and explaining the methods of performing HFU estimation employing FZI and FCM methods in this study, the investigation's limitations are presented to clarify and better understand the conditions under which the results will be interpreted.

1. Only the coring data of an exploratory well in the Asmari Formation was employed.
2. The logs of the same well were used to validate the results, but the data of the end parts of the Asmari Formation were not collected.
3. The closest well from the exploratory well (well-A) west of the Mansouri field for geological correlation is in the center of the field (well-B). The upper parts of the Asmari Formation have different deposition environments, so the dominant lithology of well-B includes sandstone and dolomite. However, the upper part of well-A contains dominant limestone, and the lower depths include sandstone and dolomite, so more exploratory and appraisal wells must be drilled to investigate this region's deposition process and geological structure.
4. Considering that core data is more reliable than drilling cutting samples and log data, due to lost circulation in some parts of the Asmari Formation, it was impossible to take cores, and the interpretation was made based on log data and drilling cuttings.
5. Employing only Fuzzy methods such as FCM has not successfully separated porosity and permeability changes.

Moreover, the methodology's advantages of this research include the following.

1. Employing the FCM technique with better data clustering in determining in-depth description and better results in HFU continuity has provided.
2. Utilizing the K-means method in the initial stages of the hydraulic flow separation based on the FZI method.
3. Using the deterministic method of FZI to determine better the rock type, each of which represents a facies with a specific range of permeability and porosity.
4. Integrating the FCM and FZI methods to better comprehend permeability and porosity relations in each rock type to define reservoir characterizations.
5. Predict the HFUs for uncored wells around the studied well.

## Results

Results of HFUs were made based on 280 core samples and well logs obtained from one of the drilled wells in the west of Mansouri field, including permeability, porosity, and formation information. The integrated analysis of FCM and FZI clustering techniques is functional with limited data to develop the porosity correlation to predict the rock type in uncored wells, especially less distance offset wells.

### Separation of HFUs based on FZI

**Figure 6:**
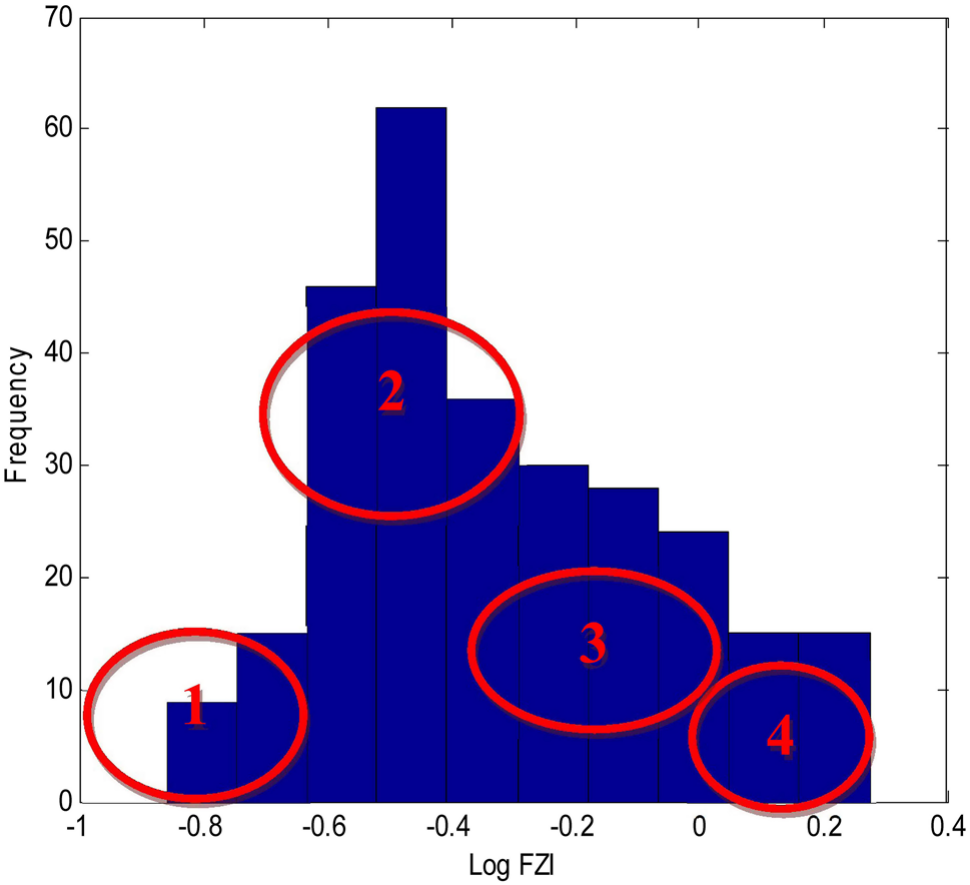
Histogram analysis on logarithmic data of the FZI.

In order to determine the number of HFUs, reservoir samples were first prepared in the laboratory, and their porosity and permeability were measured. Then, after determining the FZI of each sample, a histogram analysis was performed on the logarithmic data of the FZI, and the number of HFUs was determined based on the obtained normal distributions (Fig. 6).

**Figure 7:**
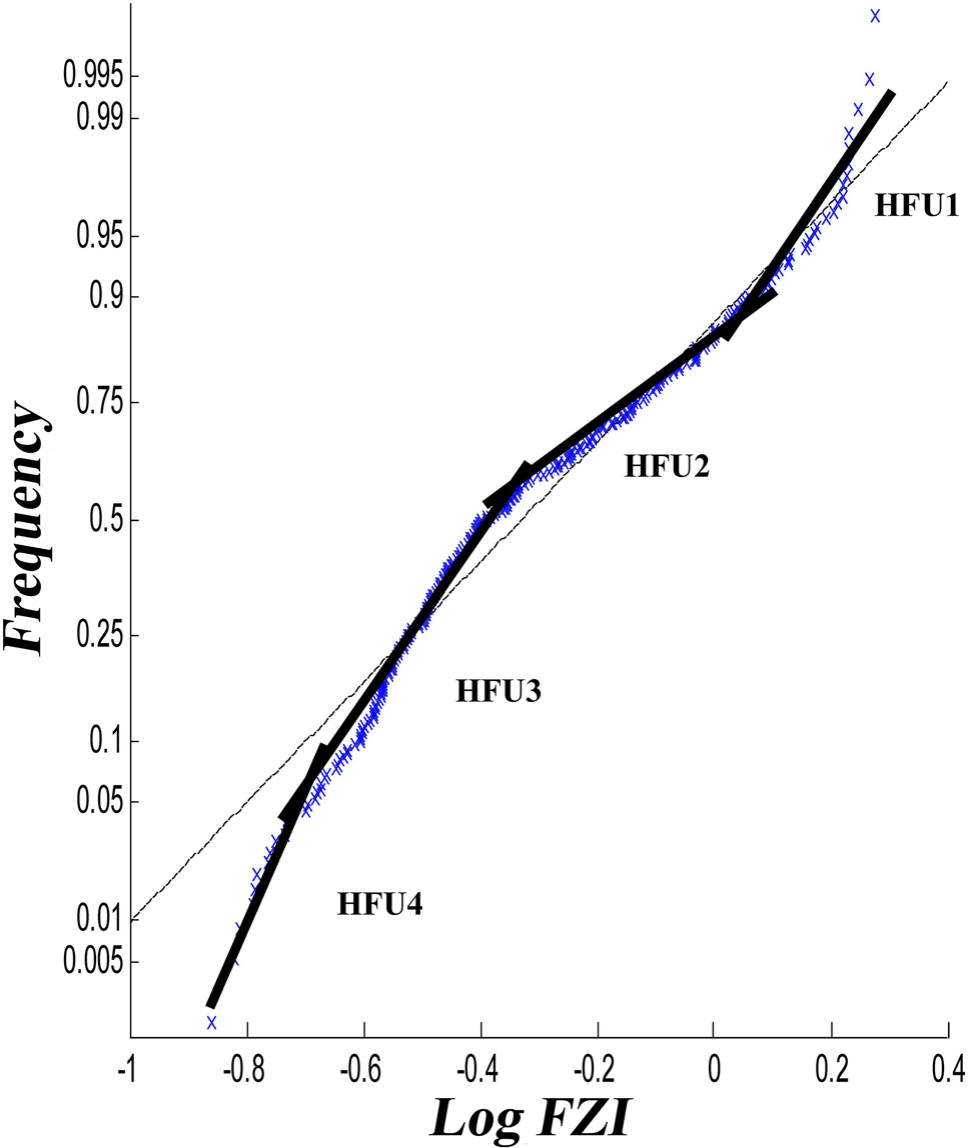
Normal probability analysis on logarithmic data of the FZI.

**Figure 8:**
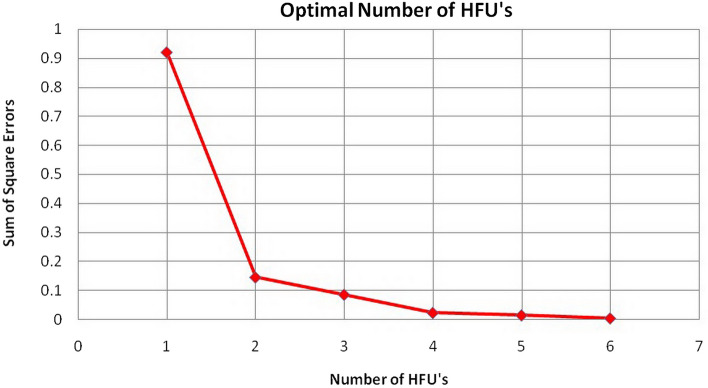
Diagram of the sum of squared errors (SSE) versus the number of HFUs.

Considering that these two methods depend on the user (this number changes according to the user's opinion and experience), the possibility of making errors in the calculations is high. The sum of squared errors (SSE) parameter was used to reduce the errors and define the HFUs' number. The working method is as follows: first, the number of categories is assumed to be 1 (HFU = 1), and the K-means cluster analysis of the data was calculated. Then, the linear regression analysis was performed on the data, and the SSE was calculated. The same way was done for several other categories. Finally, a graph of the SSE against the number of categories was drawn (Figs. 7, 8). In these graphs, from one value to the next, after considering four categories, the changes in the SSE are not noticeable and can be ignored. Then, the optimal number of HFUs is set as 4. Separation of rock-type groups using histogram and SSE has been done based on user experience. As seen in Figs. 6 and 7, it is possible to separate the rock type into six or more groups. However, increasing the number of rock groups to more than four due to a very low SSE of 0.002 will not make a noticeable interpretation change and will only be time-consuming (Fig. 8).

**Figure 9:**
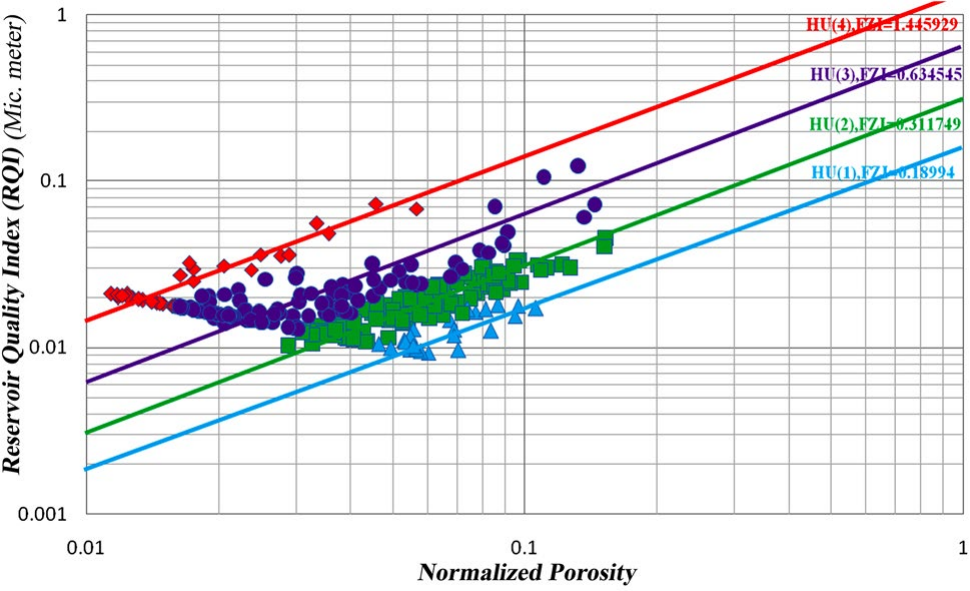
Separating of each flow unit with the FZI method.
- Table 2 shows the value of the SSE calculated according to the number of HFUs. As Table 2, the value of SSE in the presence of only one HFU is equal to 0.92, which clearly shows the inadequacy of classical methods and the existence of several fluid behaviors in the reservoir. By adding the number of HFUs, the number of SSEs decreases. However, as continue to add HFUs, the amount of SSE reduction becomes less and less; in this case, SSE is used as a criterion to determine the optimal number of HFUs in the reservoir, reaching the lowest value of SSE at 0.02 in 4 known HFU units. As seen in Fig. 8, increasing the number of HFUs causes insignificant changes in the SSE value. Figure 9 shows the normal porosity versus reservoir quality index (RQI) of all four hydraulic units. Furthermore, Table 3 shows the average FZI values for each HFU in the studied well. Calculations of the FZI values for each HFU are presented in Supplementary Table A.1 to Supplementary Table A.4, respectively.

In this study, the definition of rock type was based on parameters of porosity and permeability. Thus, each produced cluster is considered representative of a rock type. In the clustering process, each rock type will have characteristics related to statistical parameters (the minimum, maximum, mean, median, and standard deviation) and a range of porosity and permeability changes, which separates it from other types. In addition, in the cross-plots of porosity versus permeability (Fig. 9), each type is well separated from the other types, and there is no overlap. Obviously, in this case, any rock represents a facies with a specific range in terms of porosity and permeability. The HFU determination results include histogram analysis, normal probability analysis, and the SSE. These three methods are studied on core data in the following section.

- The histogram analysis results in four normal distributions representing four HFUs (Fig. 6) in which HFU 1 to 4 consists of 24, 109, 117 and 30 members, respectively.
- As the normal probability analysis results, four linear distributions are obtained, representing four HFUs; therefore, this method confirms the number of HFUs obtained from the previous step.
  In this method, normal probability analysis is performed on the logarithmic data of the FZI, and four linear distributions are obtained, representing four HFUs (Fig. 7).

**Table 2:** The value of the sum of squared errors (SSE) calculated for the number of HFUs.

No. of HFU	Sum of squared errors (SSE)
1	0.921313
2	0.144515
3	0.08462
4	0.021707
5	0.013309
6	0.002989

**Table 3:** Average FZI value for each hydraulic unit.

Hydraulic flow unit (HFU)	Flow Zone Index (FZI)
1	0.1899
2	0.3117
3	0.6345
4	1.4459

According to the results obtained from these three techniques, the SSE method is optimal for determining the number of HFUs since it is independent of the user, other methods were also able to determine the number of categories properly.

### Separation of HFUs based on FCM

**Figure 10:**
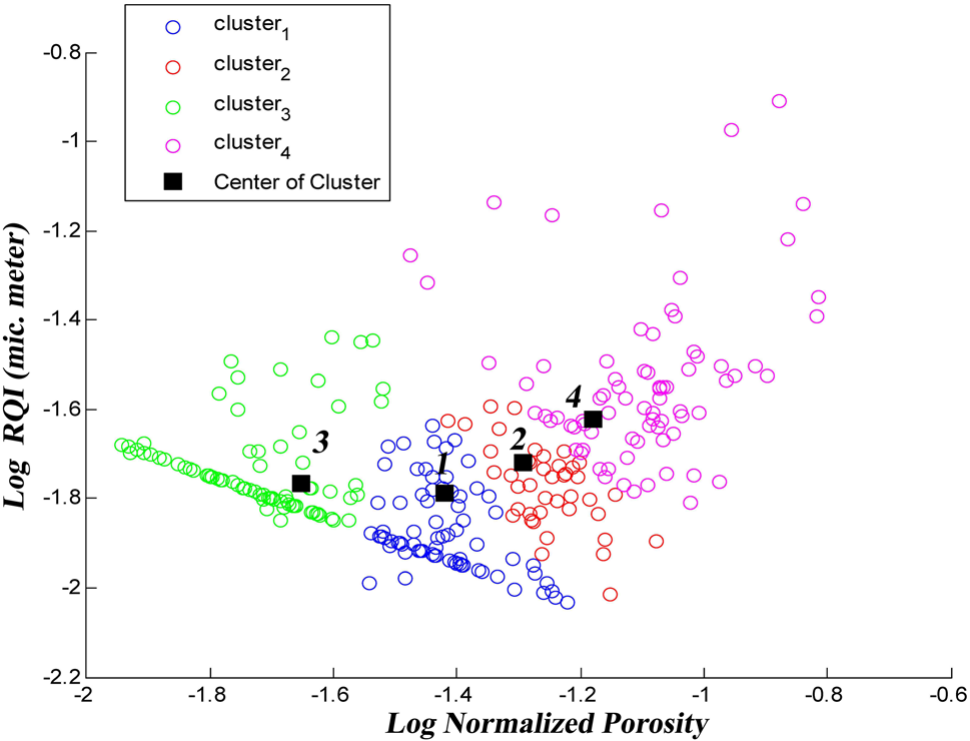
Four identical fuzzy clusters with different members utilizing the FCM method.

The FCM algorithm divides the dataset into four similar fuzzy clusters with different numbers of members. As shown in Fig. 10, each cluster is displayed with a different color, and the centers of each cluster are marked with a black square. The first to last cluster have 77, 42, 87, and 74 members, respectively.

The FCM method tries to minimize the evaluation function (J_m_) with successive iterations until a significant improvement is achieved (decreased J_m_ from 8.8759 to 3.6806 in eleven iterations and becomes constant).

Eventually, as highlights of the results:

- Discuss the dispersal of permeability and porosity variables and identify the proper reservoir quality utilizing HFUs.
- HFUs identification deals with declustering and partitioning algorithms.
  - A dissimilar must-link tough and constrained clustering procedure involves Fuzzy logic.

**Discussion**

### Comparison of rock types determination methods

Two methods of FZI and FCM are utilized to determine rock types in the study wells. In this section, the proposed approaches are compared.

Initially, both methods of rock type determination were implemented in-depth. Lateral continuity in rock types are sediment layers that initially extend laterally in all directions of a rock type are laterally continuous. HFU lateral continuity of rock units with consistent geological properties utilizing the Testerman method is used to controlling the behavior of fluid flow in pores media laterally.

**Figure 11:**
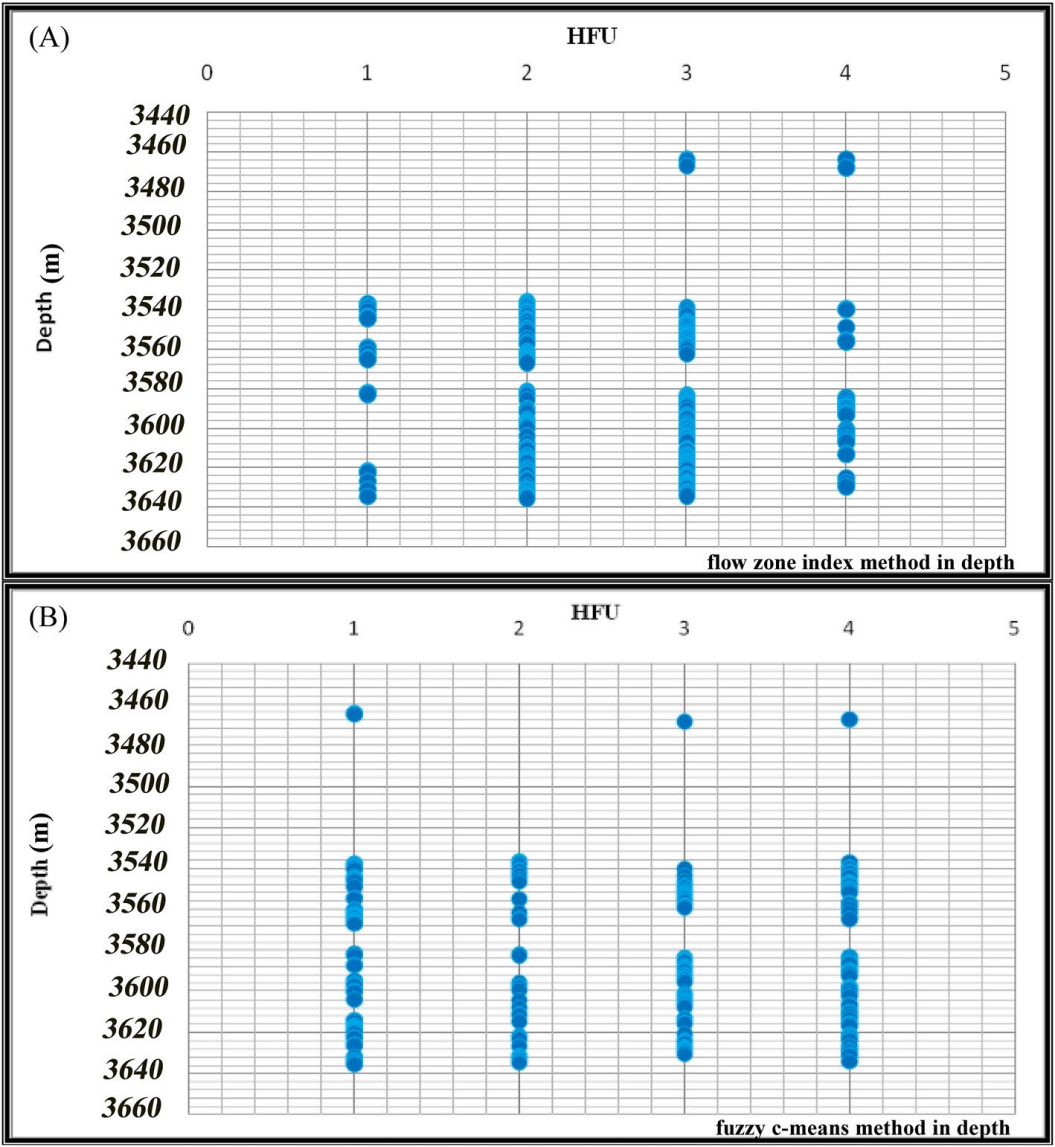
Implementation of (**a**) FZI method in depth, (**b**) FCM method in depth.

Both methods of determining rock types in depth were implemented. As shown in Fig. 11a,b, the studied well showed four HFUs. Suppose each unit has a maximum continuity number of 1, supposing four HFUs have maximum continuity. In that case, their total continuity number becomes 4, and if each of these four units has no continuity between their data, their total continuity becomes zero.

First, the data of the FZI technique have been implemented in-depth. The continuity number for the first HFU is 0.66. The second HFU is 0.79, the third HFU is 0.76, and the fourth HFU is 0.53. Finally, summing the continuity numbers of these four units, the total continuity number becomes 2.7672 (Table 4).

**Table 4:** Continuity numbers in HFUs in the FZI technique.

Flow unit no. 1	Flow unit no. 2	Flow unit no. 3	Flow unit no. 4	Total cohesion
0.6666	0.7981	0.7692	0.5333	2.7672

Then, the data obtained from the FCM technique was implemented in depth. The continuity number for the first HFU is 0.87. The second HFU is 0.61, the third HFU is 0.89, and the fourth HFU is 0.72. Finally, by summing the continuity numbers of these four units, the total continuity number is 3.1153 (Table 5).

**Table 5:** Continuity numbers in HFUs utilizing FCM technique.

Flow unit no. 1	Flow unit no. 2	Flow unit no. 3	Flow unit no. 4	Total cohesion
0.8701	0.6190	0.8965	0.7297	3.1153

According to the obtained results, the total continuity number of the FCM method is higher than the FZI in-depth, and it shows more continuity in depth.

### Changes in the porosity diagram according to permeability

**Figure 12:**
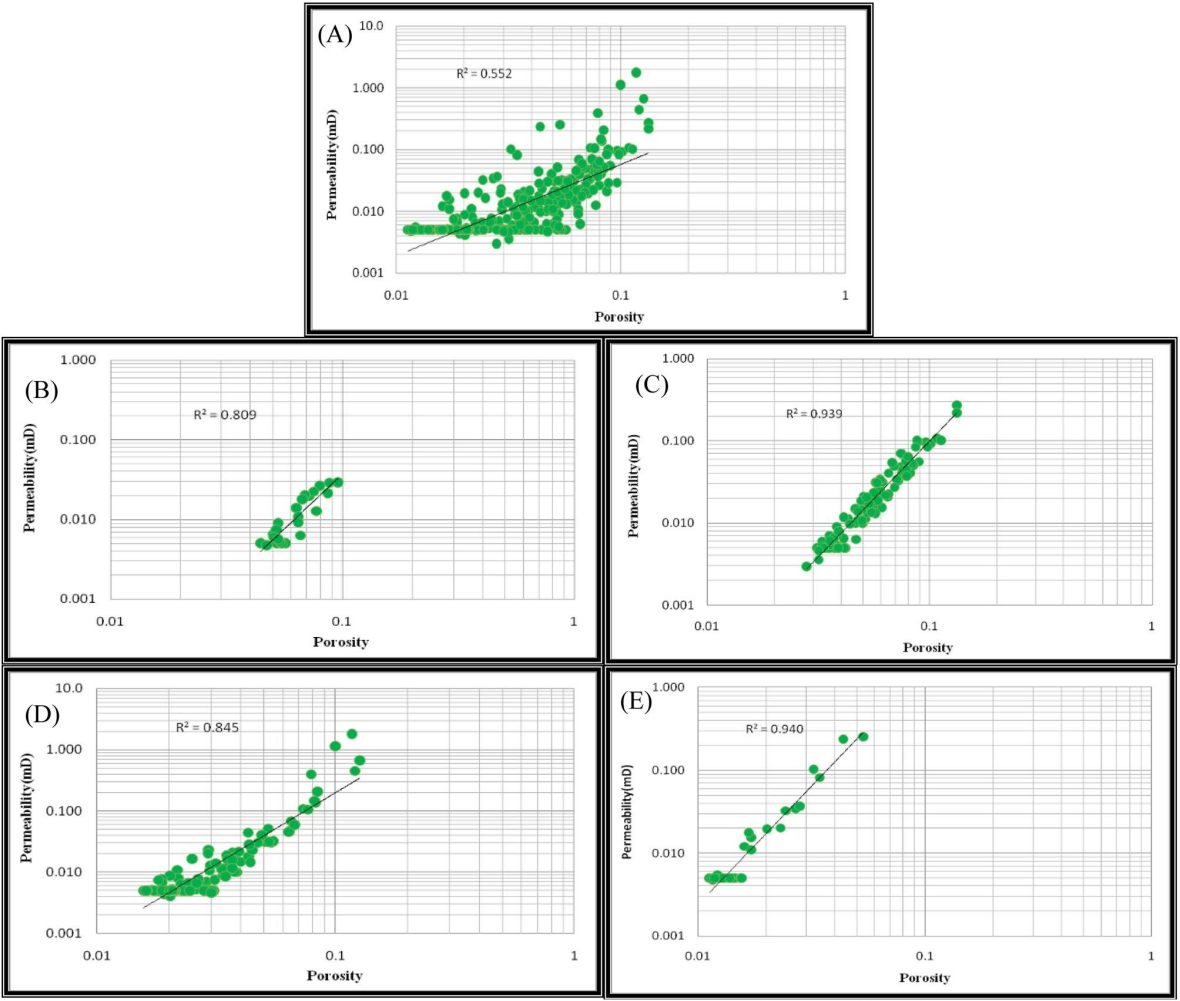
Porosity relationship with permeability using FZI method for (**A**) all samples, (**B**) unit No. 1, (**C**) unit No. 2, (**D**) unit No. 3, (**E**) unit No. 4.

**Figure 13:**
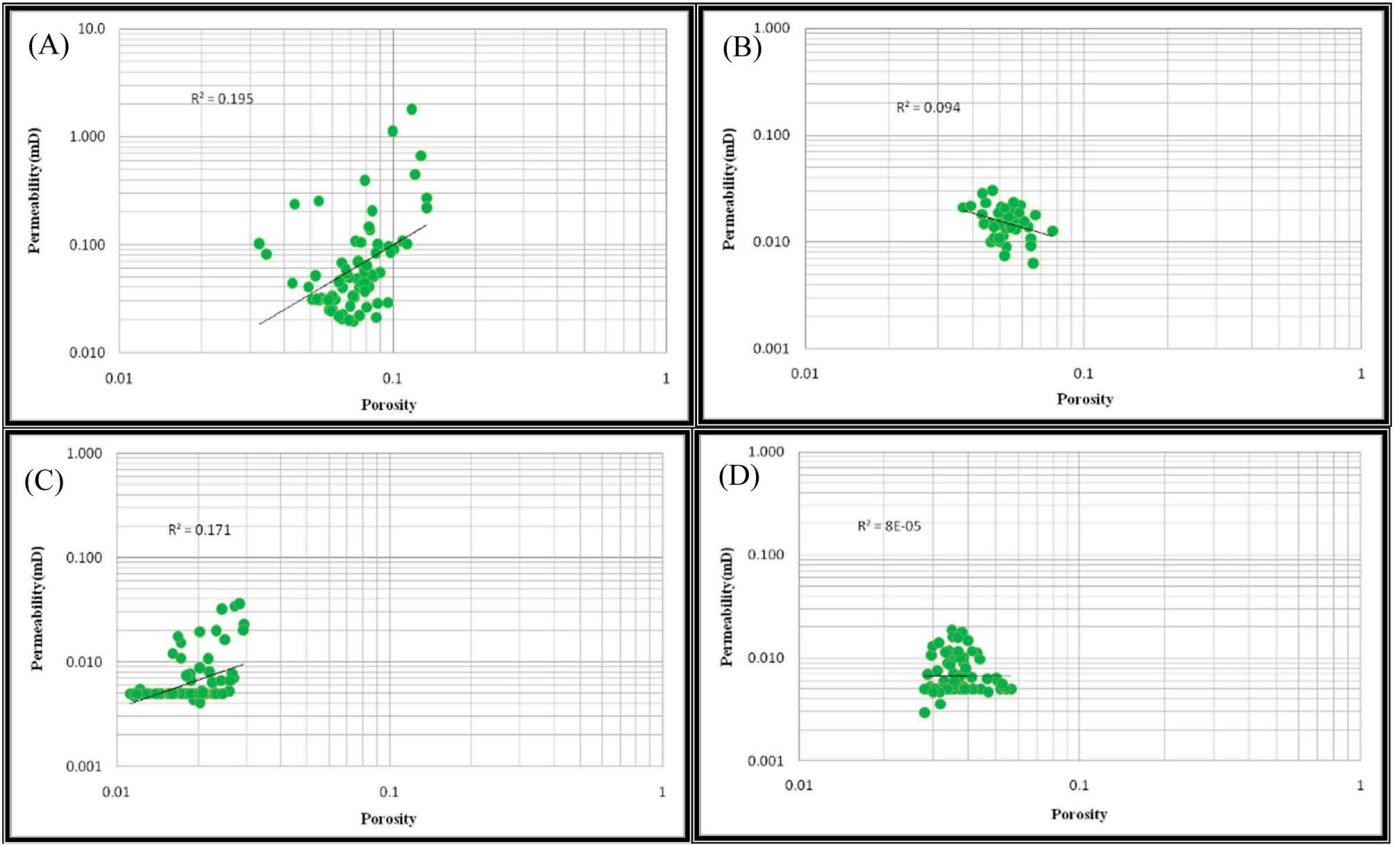
Porosity relationship with permeability for flow unit using FCM method for (**A**) unit No. 1, (**B**) unit No. 2, (**C**) unit No. 3, (**D**) unit No. 4.

Permeability–porosity diagrams in heterogeneous carbonate reservoirs are usually scattered with poor correlation (Fig. 12A) but correlate with the classification and arrangement of data regarding HFUs, demonstrating better outcomes. Permeability is observed in each HFU (Fig. 12B–E for the FZI method and also, Fig. 13A–D for the FCM method).

Tables 6 and 7 show the correlation coefficients of porosity with permeability for all samples and four units of hydraulic flow in the studied well. The correlation coefficient for all samples equals 0.552, while the correlation coefficient obtained in the FZI method for the first to last HFUs is 0.809, 0.939, 0.845, and 0.94, respectively. It indicates the improvement of the relationship between permeability and porosity in all HFUs compared to the general state for all samples.

**Table 6:** Correlation coefficients of porosity with permeability in the FZI method.

All samples	Flow unit no. 1	Flow unit no. 2	Flow unit no. 3	Flow unit no. 4
0.552	0.809	0.939	0.845	0.94

**Table 7:** Correlation coefficients of porosity with permeability in the FCM method.

All samples	Flow unit no. 1	Flow unit no. 2	Flow unit no. 3	Flow unit no. 4
0.552	0.195	0.094	0.171	0.00008

Furthermore, the correlation coefficients obtained in the FCM for the first to last HFUs are 0.195, 0.094, 0.171, and 0.00008, respectively. These results show that the correlation coefficients obtained using the FCM method in all four HFUs are lower than the general case.

**Figure 14:**
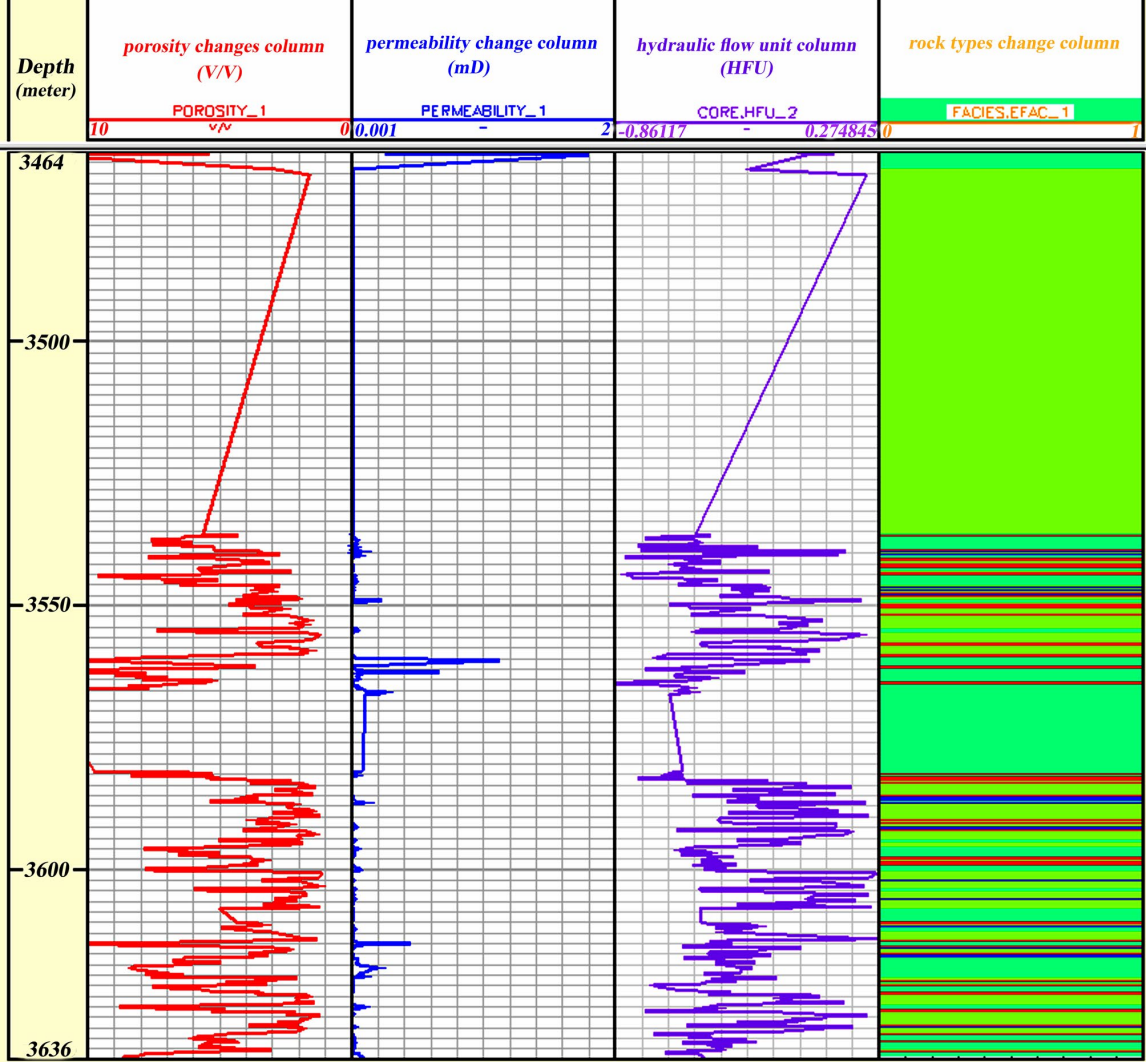
The log of changes in petrophysical parameters versus depth in the studied well includes the depth column, porosity change column, permeability change column, HFU column, and rock types change column (Rock type 1: blue, rock type 2: green, rock type 3: navy blue, and rock type 4: red). The dispersion of rock samples in rock type 2 is more than that of that of others.

Based on these results, the FZI method improved the correlation coefficients between permeability and porosity in all HFUs relative to all samples' correlation coefficients in the general states. In contrast, the FCM method did not improve the relationship between the petrophysical parameters of the reservoir in all HFUs relative to the general states while also reducing the porosity–permeability relationship. Furthermore, According to the results, the total fidelity of the FCM method is greater than the total fidelity of the FZI at depth and shows greater consistency at depth. The log of changes in petrophysical parameters versus depth in the studied well includes the depth column, porosity change column, permeability change column, HFU column, and rock types change column, shown in Fig. 14.

### Validation of results with geological facies data

Based on previous microfacies studies, four sedimentary environments of open sea, dam, lagoon and coastal environment can be distinguished for the deposition of the Asmari Formation in this field, which was deposited on a platform with a low slope. Furthermore, the deposition environment of the Oligo-Miocene era in the center of the basin was in the form of channel sand. Also, in the west of the basin, it was in the form of marine limestone^57,58^. In this study, thin sections of the Asmari Formation in the studied well-A have identified 11 general facies. Asmari formation in the Mansouri field has clastic and carbonate components. Therefore, examining existing thin sections has identified two general facies: carbonate-evaporitic facies and siliceous-clastic petrofacies. In order to validate the results, the siliceous petrofacies data is used with the assumption that the FZIs are not necessarily related to the facies and that different facies can be placed inside a specific flow unit. Based on the results of this study, porosity, and permeability in determined flow units show a good correlation coefficient. Therefore, in this way, different cavity systems with different petrophysical characteristics can be separated in the studied well in the Asmari Formation, and the facies with the best reservoir conditions can be determined.

**Figure 15:**
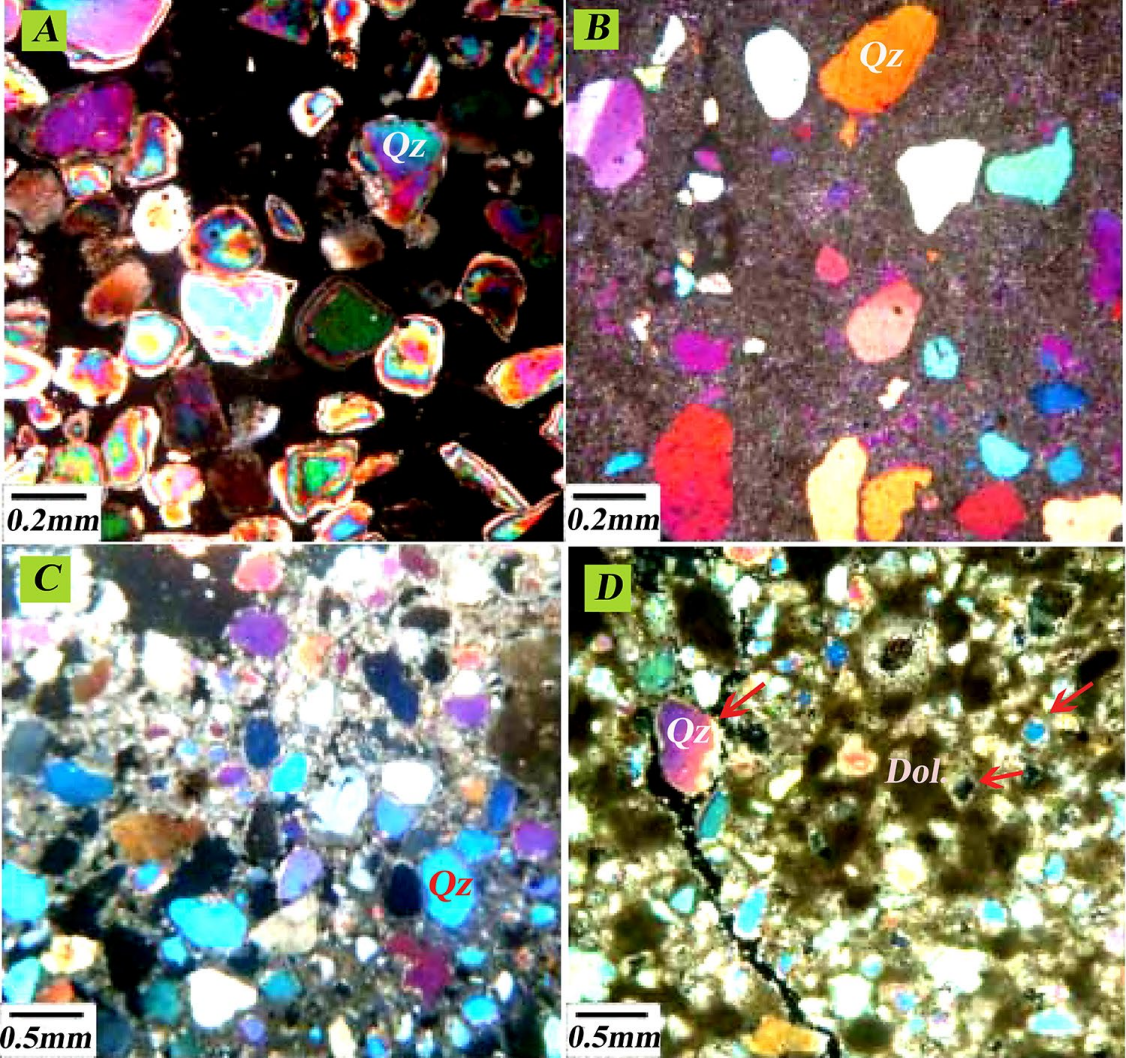
Drilling sample thin sections of the Asmari Formation sandstone zones in Mansouri field include (**a**) without cement sandstone due to migrating hydrocarbons before cementing (loose quartz grain/medium-coarse grain sub-rounded-rounded (XPL)), (**b**) dissolution in sandstones (quartz/ medium-coarse grain, subangular), (**c**) dolomite cement in sandstones (unmatured quartz/ fine grain-coarse grain/sub-rounded-subangular), (**d**) dolomite dissolution in sandstones (quartz grain/ medium-coarse grain, subrounded).

Among the designated flow units, 3 and 4 have the best flow units with high reservoir quality and permeability. On the other hand, according to the depth related to flow units 3 and 4, most of the facies that exist in these depths include sandstone and dolomite facies, and this is affected by the two factors of the primary sedimentary texture and the effect of the diagenesis process on them. Processes such as dissolution, migration of hydrocarbons to the reservoir before cementation of sandstones, and dolomitization of cement have improved the quality of the reservoir in the Ahwaz sandstone section, which can be seen in the evaluated thin section (Fig. 15). As a result, the determined flow units are affected by diagenesis processes and the type of porosity created by these processes.

### Validation between flow units and well logs

**Figure 16:**
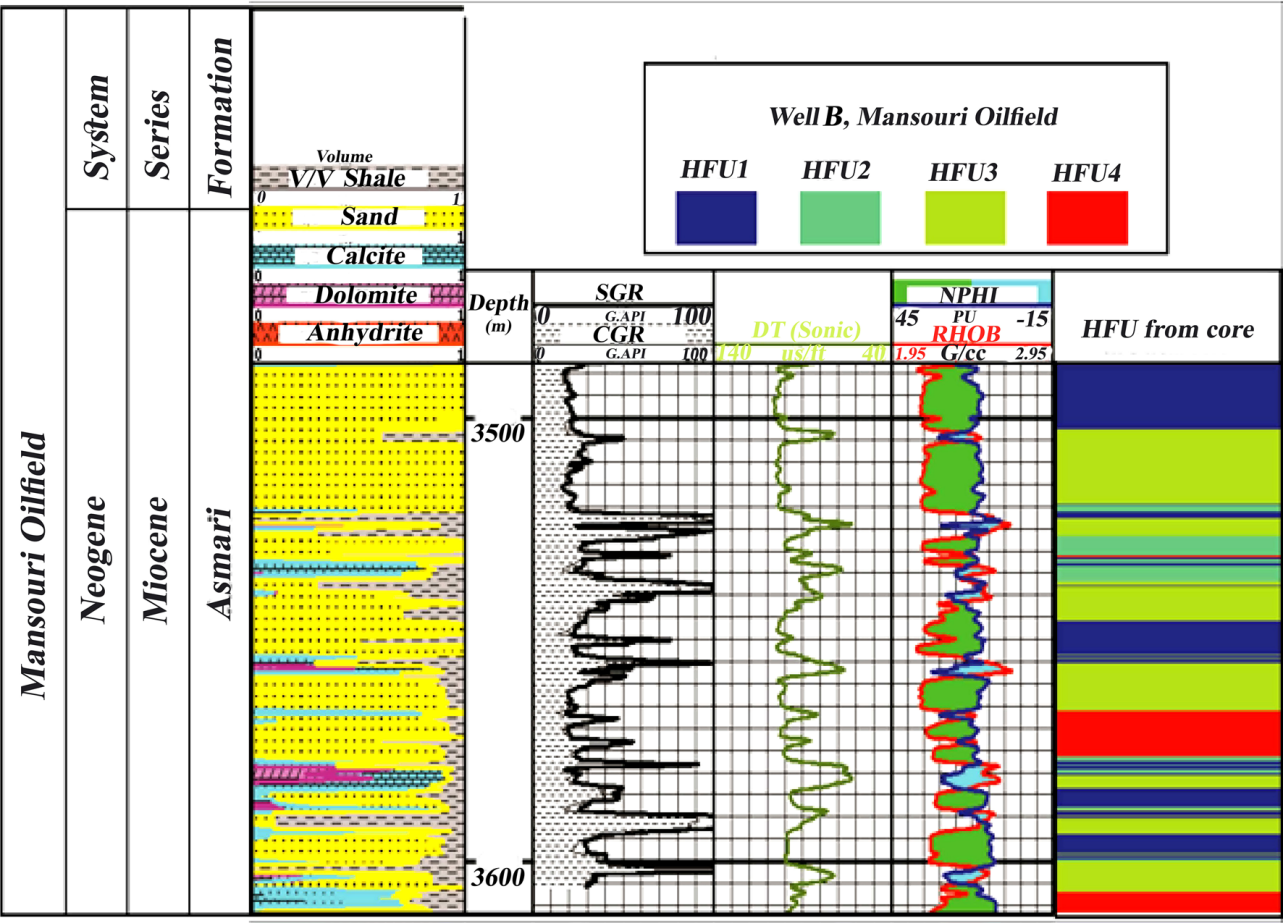
Relationship between HFUs and neutron log in the well-B of the Asmari Formation, central Mansouri oilfield. In terms of lithology, the most similarity is with Well-A in the west of the Mansoori field in the lower sandstone and dolomite zones. According to the classification of HFUs in well-B, HFU 1 (blue) and HFU 2 (blue-green) have a very low reservoir quality, HFU 3 (pale green) has the best reservoir quality and HFU 4 (red) has a low reservoir quality.

Flow units and porosity logs have been compared for additional studies in well-B in the central Mansouri field. For this purpose, the location of each flow unit in this well was determined by grouping logs and drilling cutting data in this well. According to the average data of porosity and permeability, it is concluded that the facies with pale green and red colors (HFU 3 and HFU 4) have the highest amount of porosity and permeability. As a result, they have the best reservoir quality. Finally, by comparing the porosity logs (neutron, density, and sonic) and the current unit obtained from the cutting data, it was found that the locations of the reservoir where the flow units 3 and 4 are more extended almost coincide with the locations where Neutron, density and sonic logs show more favorable conditions in terms of the reservoir (Fig. 16, Table 8). Meanwhile, in these depths, we see the presence of sandstone and dolomite and fewer limestone facies. Furthermore, a dissimilar condition is depicted in Fig. 14 for the results of HFUs in upper depths. Thus, more drilling wells are needed for lithological correlation in the Asmari Formation.

**Table 8:** Average petrophysical characteristics of each flow unit of the Asmari Formation in well-B located in the center of Mansouri field (the best HFUs are units 3 and 4 located in sandstone zones).

HFU (Well-B) Asmari Formation	Porosity (%) (mean)	Permeability (MD) (mean)	Log FZI (mean)
1	0.1616	1.0852	− 0.4247
2	0.1502	21.0148	0.1060
3	0.1511	298.7358	0.7815
4	0.0862	160.9234	0.7514

## Conclusions

Regarding the determination of HFUs on 280 core samples of the Mansouri field, using two different methods, the obtained result can be summarized as follows:

- Four HFUs were determined for the studied data after classifying FZI values by normal probability analysis, histogram analysis, and SSE. However, all three methods introduced four HFUs among the data; the SSE method can be optimal for determining the number of HFUs due to lower error rates than the other two techniques. Average FZI values were calculated at 0.19 for the first unit, 0.31 for the second, 0.63 for the third, and 1.44 for the fourth HFU in the studied well-A. The FCM clustering method divides the dataset into four identical fuzzy clusters with different member numbers by minimizing the evaluation function (J_m_).
- According to the results obtained from implementing FZI and FCM methods in depth and applying the correlation coefficient index, the total correlation of the FZI technique is 2.7672, and in the FCM method is 3.1153. Although the FCM method shows more consistency in-depth than the FZI method, the FZI method improves and enhances the correlation coefficients of porosity relation with permeability in each HFU. It means that although the Fuzzy classifier was successful in data clustering, it could not improve reservoir property relation as a sole numerical method. In contrast, the FCM method's correlation coefficient in all HFUs is lower than in the general state.
- In analyzing permeability and porosity graphs, samples with higher FZI have higher permeability at a fixed porosity so that FZI values can be a good criterion for pore correlations. Different reservoir rock samples' porosity and permeability values are highly dispersed, and using HFUs dramatically improves the relationship between these two parameters. In this research, the correlation coefficient between porosity and permeability ranged from 0.552 for all samples to 0.809 in the first, 0.939 in the second, 0.845 in the third, and 0.94 increase in the fourth HFU since samples with similar flow characteristics were placed in a single HFU. It shows a considerable improvement via HFU separation.
- In determining HFUs with the FZI method, HFUs 3 and 4 have high reservoir quality. Determined HFUs are affected by diagenesis processes and the type of porosity created, so flow units 3 and 4 are more consistent with sandstone and dolomite facies. The reason for creating these facies is processes such as fracturing, dissolution, dolomite cement, and the migration time of hydrocarbons before the cementation that has increased the reservoir quality of these facies. At reservoir depths where high-quality flow units extend, neutron, density, and sonic logs also show good reservoir quality. Therefore, it is possible to use HFUs to determine rock types in cored wells and generalize the results to coreless wells.

Furthermore, as the recommendation of this work, deeper formations of this field, such as the Bangestan Group (Sarvak and Ilam Formations), can also study the HFU using other clustering methods, such as K-means, Hierarchical, Gaussian, and fractal geometry. Also, considering the use of fractal methods in the studies of interval velocity and formation pressure in recent years, it is suggested that fractal methods such as HFU-Volume be used to verify and validate models for future studies.

### Supplementary Information

Supplementary Information.

## Data Availability

The following datasets generated and/or analyzed during the current study are available in the Mahmoud Memariani repository, doi: 10.13140/RG.2.2.19913.31847. The other datasets generated and/or analyzed during the current study are not publicly available due to not permitted to share by National Iranian Oil Company Exploration Directorate (NIOC-EXP) request but are available from the corresponding author on reasonable request.

## Abbreviations

ANN: Artificial neural network
COUCSI: Spontaneous absorption
FCM: Fuzzy c-means
FZI: Flow zone index
HFU: Hydraulic flow unit
J_m_: Evaluation function
RQI: Reservoir Quality Index
SCAL: Specific core analysis
SSE: Sum of squared errors
