# Supplementary material for: Hydraulic flow unit and rock types of the Asmari Formation, an application of flow zone index and fuzzy C-means clustering methods
Source: Sci Rep. 2024 Feb 29;14:5003. doi: 10.1038/s41598-024-55741-y (PMC10904754; doi:10.1038/s41598-024-55741-y)
Supplement: Supplementary file 1 — Supplementary Information. [file 41598_2024_55741_MOESM1_ESM.docx]

**Hydraulic flow unit and rock types of the Asmari Formation, an application of Flow zone index and Fuzzy C-means clustering methods**

**Seyedeh Hajar Eftekhari** [**^1^**](file:///E:\Ph.D\06-%20Articles\01-%20Petroleum%20Exploration%20Springer\01-%20Electrical%20facies\03-%20Article\01-%20Electical%20Facies%20Springer%20PEPT.docx#Department_of_Earth_Sciences)**, Mahmoud Memariani** [**^1^**](file:///E:\Ph.D\06-%20Articles\01-%20Petroleum%20Exploration%20Springer\01-%20Electrical%20facies\03-%20Article\01-%20Electical%20Facies%20Springer%20PEPT.docx#Department_of_Earth_Sciences)**^,^*****, Zahra Maleki** [**^1^**](file:///E:\Ph.D\06-%20Articles\01-%20Petroleum%20Exploration%20Springer\01-%20Electrical%20facies\03-%20Article\01-%20Electical%20Facies%20Springer%20PEPT.docx#Department_of_Earth_Sciences)**, Mohsen Aleali** [**^1^**](file:///E:\Ph.D\06-%20Articles\01-%20Petroleum%20Exploration%20Springer\01-%20Electrical%20facies\03-%20Article\01-%20Electical%20Facies%20Springer%20PEPT.docx#Department_of_Earth_Sciences)**,**

**Pooria Kianoush** [**^2^**](file:///E:\Ph.D\06-%20Articles\01-%20Petroleum%20Exploration%20Springer\01-%20Electrical%20facies\03-%20Article\01-%20Electical%20Facies%20Springer%20PEPT.docx#Department_of_Petroleum_and_Mining_Engin)**^,^** [**^3^**](file:///E:\Ph.D\06-%20Articles\01-%20Petroleum%20Exploration%20Springer\01-%20Electrical%20facies\03-%20Article\01-%20Electical%20Facies%20Springer%20PEPT.docx#National_ranian_Oil_Company)

*1 - Department of Earth Sciences, Science and Research Branch, Islamic Azad University, Tehran, Iran*

*2 - Department of Petroleum and Mining Engineering, South Tehran Branch, Islamic Azad University, Tehran, Iran*

*3 - National Iranian Oil Company, Exploration Directorate (NIOC-EXP), Tehran, Iran*

Corresponding author. *E-mail address:* [*Mahmoud.memariani@gmail.com*](mailto:Mahmoud.memariani@gmail.com) (M. Memariani).

**Supplementary material**

**A: Calculations per hydraulic flow unit**

| **FZI** | **RQI** | **φ_z_** | **K** | **φ** |
| --- | --- | --- | --- | --- |
| 0.217128 | 0.014648 | 0.067462 | 0.014 | 0.063198 |
| 0.185614 | 0.012794 | 0.068926 | 0.011 | 0.064482 |
| 0.172042 | 0.011852 | 0.068893 | 0.009 | 0.064453 |
| 0.177558 | 0.009738 | 0.054842 | 0.005 | 0.051991 |
| 0.151402 | 0.012687 | 0.083797 | 0.013 | 0.077318 |
| 0.212371 | 0.011237 | 0.052912 | 0.006 | 0.050253 |
| 0.154558 | 0.009313 | 0.060259 | 0.005 | 0.056834 |
| 0.163056 | 0.01729 | 0.106038 | 0.029 | 0.095872 |
| 0.163734 | 0.015521 | 0.094794 | 0.021 | 0.086586 |
| 0.227787 | 0.010552 | 0.046325 | 0.005 | 0.044274 |
| 0.207443 | 0.018029 | 0.086912 | 0.026 | 0.079963 |
| 0.185822 | 0.01791 | 0.096383 | 0.029 | 0.08791 |
| 0.165077 | 0.009512 | 0.057623 | 0.005 | 0.054484 |
| 0.137667 | 0.009694 | 0.070417 | 0.006 | 0.065784 |
| 0.212788 | 0.01642 | 0.077166 | 0.020 | 0.071638 |
| 0.232184 | 0.012938 | 0.055722 | 0.009 | 0.052781 |
| 0.216507 | 0.011849 | 0.05473 | 0.007 | 0.05189 |
| 0.173463 | 0.009816 | 0.05659 | 0.005 | 0.053559 |
| 0.202049 | 0.010789 | 0.053397 | 0.006 | 0.05069 |
| 0.209661 | 0.017045 | 0.081296 | 0.022 | 0.075184 |
| 0.228771 | 0.016932 | 0.074015 | 0.020 | 0.068914 |
| 0.183893 | 0.01026 | 0.055794 | 0.006 | 0.052846 |
| 0.200278 | 0.009908 | 0.049472 | 0.005 | 0.04714 |
| 0.225228 | 0.016177 | 0.071825 | 0.018 | 0.067012 |

**Supplementary Table A. 1** Classification of samples in hydraulic flow unit 1.

| **FZI** | **RQI** | **φ_z_** | **K** | **φ** |
| --- | --- | --- | --- | --- |
| 0.303152 | 0.018051 | 0.059543 | 0.019 | 0.056197 |
| 0.336037 | 0.018424 | 0.054827 | 0.018 | 0.051977 |
| 0.357426 | 0.016051 | 0.044908 | 0.011 | 0.042978 |
| 0.299601 | 0.024713 | 0.082488 | 0.047 | 0.076202 |
| 0.320061 | 0.020415 | 0.063783 | 0.025 | 0.059959 |
| 0.282344 | 0.022996 | 0.081446 | 0.040 | 0.075312 |
| 0.326306 | 0.011857 | 0.036338 | 0.005 | 0.035064 |
| 0.271302 | 0.014699 | 0.054178 | 0.011 | 0.051394 |
| 0.394689 | 0.013404 | 0.033961 | 0.006 | 0.032845 |
| 0.284772 | 0.011344 | 0.039835 | 0.005 | 0.038309 |
| 0.247716 | 0.010843 | 0.04377 | 0.005 | 0.041934 |
| 0.303825 | 0.018631 | 0.06132 | 0.020 | 0.057777 |
| 0.262019 | 0.015989 | 0.061023 | 0.015 | 0.057514 |
| 0.271338 | 0.021163 | 0.077994 | 0.033 | 0.072351 |
| 0.381888 | 0.020317 | 0.053201 | 0.021 | 0.050514 |
| 0.281219 | 0.021581 | 0.076742 | 0.034 | 0.071272 |
| 0.329206 | 0.020452 | 0.062126 | 0.025 | 0.058492 |
| 0.256051 | 0.017757 | 0.069348 | 0.021 | 0.06485 |
| 0.390119 | 0.016757 | 0.042954 | 0.012 | 0.041184 |
| 0.235823 | 0.011561 | 0.049025 | 0.006 | 0.046734 |
| 0.288299 | 0.011389 | 0.039505 | 0.005 | 0.038004 |
| 0.288873 | 0.011593 | 0.040131 | 0.005 | 0.038582 |
| 0.387729 | 0.012543 | 0.032349 | 0.005 | 0.031336 |
| 0.350797 | 0.01214 | 0.034607 | 0.005 | 0.033449 |
| 0.291277 | 0.012499 | 0.042911 | 0.007 | 0.041145 |
| 0.317669 | 0.025366 | 0.07985 | 0.048 | 0.073945 |
| 0.304215 | 0.019129 | 0.06288 | 0.022 | 0.05916 |
| 0.320642 | 0.01179 | 0.03677 | 0.005 | 0.035466 |
| 0.323244 | 0.011821 | 0.03657 | 0.005 | 0.03528 |
| 0.344846 | 0.012073 | 0.035009 | 0.005 | 0.033824 |
| 0.345046 | 0.013032 | 0.03777 | 0.006 | 0.036395 |
| 0.264504 | 0.013987 | 0.052881 | 0.010 | 0.050225 |
| 0.293684 | 0.031329 | 0.106677 | 0.096 | 0.096394 |
| 0.272656 | 0.022922 | 0.08407 | 0.041 | 0.077551 |
| 0.251041 | 0.024702 | 0.0984 | 0.055 | 0.089585 |
| 0.248956 | 0.022169 | 0.089048 | 0.041 | 0.081767 |
| 0.356273 | 0.026554 | 0.074533 | 0.050 | 0.069363 |
| 0.328068 | 0.028153 | 0.085815 | 0.064 | 0.079032 |
| 0.285729 | 0.015854 | 0.055486 | 0.013 | 0.052569 |
| 0.262636 | 0.024263 | 0.092383 | 0.050 | 0.08457 |
| 0.288623 | 0.023891 | 0.082777 | 0.044 | 0.076449 |
| 0.266659 | 0.029899 | 0.112126 | 0.091 | 0.100821 |
| 0.25733 | 0.031236 | 0.121385 | 0.107 | 0.108246 |
| 0.293744 | 0.044959 | 0.153055 | 0.272 | 0.132739 |
| 0.265072 | 0.040454 | 0.152616 | 0.220 | 0.132408 |
| 0.235575 | 0.029854 | 0.126727 | 0.102 | 0.112474 |
| 0.269108 | 0.029189 | 0.108466 | 0.085 | 0.097852 |
| 0.24206 | 0.015766 | 0.065132 | 0.015 | 0.06115 |
| 0.272144 | 0.024871 | 0.091388 | 0.053 | 0.083736 |
| 0.27452 | 0.01121 | 0.040834 | 0.005 | 0.039232 |
| 0.281162 | 0.011297 | 0.04018 | 0.005 | 0.038628 |
| 0.295912 | 0.011486 | 0.038816 | 0.005 | 0.037366 |
| 0.38945 | 0.012561 | 0.032253 | 0.005 | 0.031245 |
| 0.382599 | 0.014049 | 0.03672 | 0.007 | 0.03542 |
| 0.388932 | 0.012555 | 0.032282 | 0.005 | 0.031272 |
| 0.252645 | 0.010912 | 0.043191 | 0.005 | 0.041403 |
| 0.301848 | 0.017866 | 0.059189 | 0.018 | 0.055882 |
| 0.277765 | 0.023722 | 0.085404 | 0.045 | 0.078684 |
| 0.261237 | 0.019567 | 0.074901 | 0.027 | 0.069682 |
| 0.340635 | 0.02235 | 0.065613 | 0.031 | 0.061573 |
| 0.363928 | 0.019037 | 0.052309 | 0.018 | 0.049709 |
| 0.263194 | 0.018439 | 0.070058 | 0.023 | 0.065471 |
| 0.382473 | 0.015259 | 0.039896 | 0.009 | 0.038365 |
| 0.339542 | 0.013446 | 0.039602 | 0.007 | 0.038093 |
| 0.367272 | 0.012454 | 0.033909 | 0.005 | 0.032797 |
| 0.320668 | 0.01179 | 0.036768 | 0.005 | 0.035464 |
| 0.297317 | 0.014531 | 0.048872 | 0.010 | 0.046595 |
| 0.366129 | 0.017801 | 0.04862 | 0.015 | 0.046366 |
| 0.331112 | 0.028131 | 0.08496 | 0.063 | 0.078307 |
| 0.373331 | 0.030239 | 0.080997 | 0.069 | 0.074928 |
| 0.317551 | 0.020087 | 0.063257 | 0.024 | 0.059494 |
| 0.323629 | 0.018789 | 0.058056 | 0.020 | 0.054871 |
| 0.324016 | 0.016962 | 0.052348 | 0.015 | 0.049744 |
| 0.321781 | 0.014787 | 0.045954 | 0.010 | 0.043935 |
| 0.334056 | 0.01226 | 0.0367 | 0.005 | 0.035401 |
| 0.343153 | 0.013181 | 0.038411 | 0.007 | 0.03699 |
| 0.370506 | 0.01926 | 0.051982 | 0.019 | 0.049413 |
| 0.338185 | 0.032896 | 0.097273 | 0.097 | 0.08865 |
| 0.269227 | 0.014113 | 0.052421 | 0.010 | 0.04981 |
| 0.349096 | 0.012121 | 0.034721 | 0.005 | 0.033556 |
| 0.346069 | 0.014114 | 0.040784 | 0.008 | 0.039186 |
| 0.271838 | 0.018407 | 0.067713 | 0.022 | 0.063419 |
| 0.385322 | 0.028055 | 0.072809 | 0.054 | 0.067868 |
| 0.369602 | 0.023565 | 0.063756 | 0.034 | 0.059935 |
| 0.329889 | 0.027987 | 0.084837 | 0.062 | 0.078203 |
| 0.354758 | 0.010207 | 0.028771 | 0.003 | 0.027967 |
| 0.337864 | 0.016878 | 0.049954 | 0.014 | 0.047577 |
| 0.321052 | 0.010536 | 0.032817 | 0.004 | 0.031774 |
| 0.248206 | 0.015047 | 0.060624 | 0.013 | 0.057159 |
| 0.313233 | 0.017723 | 0.056581 | 0.017 | 0.053551 |
| 0.348731 | 0.012915 | 0.037035 | 0.006 | 0.035713 |
| 0.379642 | 0.023058 | 0.060737 | 0.031 | 0.057259 |
| 0.350095 | 0.033691 | 0.096233 | 0.101 | 0.087786 |
| 0.380345 | 0.030504 | 0.0802 | 0.070 | 0.074246 |
| 0.299131 | 0.014948 | 0.049972 | 0.011 | 0.047594 |
| 0.277935 | 0.011255 | 0.040494 | 0.005 | 0.038918 |
| 0.366876 | 0.012024 | 0.032775 | 0.005 | 0.031735 |
| 0.284751 | 0.017735 | 0.062283 | 0.019 | 0.058631 |
| 0.363869 | 0.023247 | 0.063888 | 0.033 | 0.060052 |
| 0.26947 | 0.015609 | 0.057923 | 0.014 | 0.054752 |
| 0.340298 | 0.020211 | 0.059393 | 0.023 | 0.056063 |
| 0.370996 | 0.022872 | 0.06165 | 0.031 | 0.05807 |
| 0.279445 | 0.014644 | 0.052402 | 0.011 | 0.049793 |
| 0.250771 | 0.021484 | 0.08567 | 0.037 | 0.07891 |
| 0.313365 | 0.026641 | 0.085017 | 0.056 | 0.078355 |
| 0.322271 | 0.028073 | 0.087109 | 0.064 | 0.080129 |
| 0.326987 | 0.030943 | 0.094632 | 0.084 | 0.086451 |
| 0.357847 | 0.019699 | 0.05505 | 0.021 | 0.052178 |
| 0.352012 | 0.024617 | 0.069933 | 0.040 | 0.065362 |

**Supplementary Table A. 2.** Classification of samples in hydraulic flow unit 2.

| **FZI** | **RQI** | **φ_z_** | **K** | **φ** |
| --- | --- | --- | --- | --- |
| 0.821896 | 0.070335 | 0.085577 | 0.396 | 0.078831 |
| 0.931807 | 0.123413 | 0.132445 | 1.807 | 0.116955 |
| 0.523308 | 0.015474 | 0.02957 | 0.007 | 0.028721 |
| 0.568126 | 0.03125 | 0.055006 | 0.052 | 0.052138 |
| 0.462246 | 0.03216 | 0.069573 | 0.068 | 0.065047 |
| 0.395756 | 0.012627 | 0.031906 | 0.005 | 0.030919 |
| 0.640829 | 0.014785 | 0.023072 | 0.005 | 0.022552 |
| 0.586446 | 0.016143 | 0.027527 | 0.007 | 0.02679 |
| 0.615904 | 0.023637 | 0.038378 | 0.021 | 0.03696 |
| 0.43232 | 0.012997 | 0.030063 | 0.005 | 0.029185 |
| 0.634629 | 0.023 | 0.036241 | 0.019 | 0.034974 |
| 0.482788 | 0.015491 | 0.032087 | 0.008 | 0.031089 |
| 0.48619 | 0.017655 | 0.036313 | 0.011 | 0.035041 |
| 0.54344 | 0.02149 | 0.039545 | 0.018 | 0.038041 |
| 0.719984 | 0.015362 | 0.021336 | 0.005 | 0.020891 |
| 0.784676 | 0.015803 | 0.020139 | 0.005 | 0.019741 |
| 0.621626 | 0.01692 | 0.027219 | 0.008 | 0.026498 |
| 0.533951 | 0.014182 | 0.026561 | 0.005 | 0.025873 |
| 0.611963 | 0.014563 | 0.023797 | 0.005 | 0.023244 |
| 0.756374 | 0.015613 | 0.020642 | 0.005 | 0.020224 |
| 1.080316 | 0.017557 | 0.016252 | 0.005 | 0.015992 |
| 0.696502 | 0.015195 | 0.021817 | 0.005 | 0.021351 |
| 0.93327 | 0.016731 | 0.017927 | 0.005 | 0.017611 |
| 0.784077 | 0.015799 | 0.020149 | 0.005 | 0.019751 |
| 0.803665 | 0.015927 | 0.019818 | 0.005 | 0.019433 |
| 0.710573 | 0.031879 | 0.044864 | 0.044 | 0.042937 |
| 1.059298 | 0.017444 | 0.016468 | 0.005 | 0.016201 |
| 0.935003 | 0.016741 | 0.017905 | 0.005 | 0.01759 |
| 0.438691 | 0.013059 | 0.029768 | 0.005 | 0.028908 |
| 0.721174 | 0.016675 | 0.023122 | 0.006 | 0.022599 |
| 0.618458 | 0.014614 | 0.023629 | 0.005 | 0.023084 |
| 0.796664 | 0.015882 | 0.019935 | 0.005 | 0.019545 |
| 1.048914 | 0.017387 | 0.016577 | 0.005 | 0.016306 |
| 0.830764 | 0.016102 | 0.019382 | 0.005 | 0.019014 |
| 0.928895 | 0.016705 | 0.017984 | 0.005 | 0.017666 |
| 0.956519 | 0.105766 | 0.110574 | 1.130 | 0.099565 |
| 0.500786 | 0.072332 | 0.144437 | 0.670 | 0.126208 |
| 0.63083 | 0.014709 | 0.023317 | 0.005 | 0.022786 |
| 0.931196 | 0.016719 | 0.017954 | 0.005 | 0.017637 |
| 0.81136 | 0.015703 | 0.019354 | 0.005 | 0.018987 |
| 0.726389 | 0.015143 | 0.020847 | 0.005 | 0.020421 |
| 0.442144 | 0.013341 | 0.030173 | 0.005 | 0.029289 |
| 0.633691 | 0.014717 | 0.023224 | 0.005 | 0.022697 |
| 0.461424 | 0.019175 | 0.041556 | 0.015 | 0.039898 |
| 0.581997 | 0.021278 | 0.03656 | 0.016 | 0.03527 |
| 0.419758 | 0.023742 | 0.05656 | 0.031 | 0.053532 |
| 1.00398 | 0.02224 | 0.022152 | 0.011 | 0.021672 |
| 0.564041 | 0.014179 | 0.025138 | 0.005 | 0.024522 |
| 0.570881 | 0.014235 | 0.024935 | 0.005 | 0.024328 |
| 0.855366 | 0.019098 | 0.022327 | 0.008 | 0.02184 |
| 0.42507 | 0.012925 | 0.030407 | 0.005 | 0.02951 |
| 0.44182 | 0.016719 | 0.03784 | 0.010 | 0.036461 |
| 1.006725 | 0.020751 | 0.020613 | 0.009 | 0.020196 |
| 0.766925 | 0.014993 | 0.01955 | 0.004 | 0.019175 |
| 0.461188 | 0.024609 | 0.053359 | 0.031 | 0.050656 |
| 0.874515 | 0.016376 | 0.018726 | 0.005 | 0.018382 |
| 0.729368 | 0.016741 | 0.022953 | 0.006 | 0.022438 |
| 0.44989 | 0.020286 | 0.045091 | 0.018 | 0.043146 |
| 0.401069 | 0.016093 | 0.040126 | 0.010 | 0.038578 |
| 0.457304 | 0.016627 | 0.036359 | 0.010 | 0.035084 |
| 0.414875 | 0.024027 | 0.057913 | 0.032 | 0.054743 |
| 0.618686 | 0.018855 | 0.030476 | 0.011 | 0.029575 |
| 0.666073 | 0.016473 | 0.024732 | 0.007 | 0.024135 |
| 0.521806 | 0.018439 | 0.035338 | 0.012 | 0.034132 |
| 1.062884 | 0.020186 | 0.018992 | 0.008 | 0.018638 |
| 0.896105 | 0.016508 | 0.018422 | 0.005 | 0.018089 |
| 0.6079 | 0.014531 | 0.023904 | 0.005 | 0.023346 |
| 1.0041 | 0.017323 | 0.017252 | 0.005 | 0.016959 |
| 0.986015 | 0.018799 | 0.019066 | 0.007 | 0.018709 |
| 0.922586 | 0.027856 | 0.030194 | 0.023 | 0.029309 |
| 0.673591 | 0.02072 | 0.030761 | 0.013 | 0.029843 |
| 0.461549 | 0.016135 | 0.034959 | 0.009 | 0.033778 |
| 0.807181 | 0.01595 | 0.01976 | 0.005 | 0.019377 |
| 0.534495 | 0.018388 | 0.034402 | 0.011 | 0.033258 |
| 0.569495 | 0.023308 | 0.040928 | 0.022 | 0.039319 |
| 0.484992 | 0.022627 | 0.046654 | 0.023 | 0.044574 |
| 0.407819 | 0.012751 | 0.031267 | 0.005 | 0.030319 |
| 0.419693 | 0.016378 | 0.039024 | 0.010 | 0.037558 |
| 0.443056 | 0.060568 | 0.136704 | 0.447 | 0.120264 |
| 0.87352 | 0.026199 | 0.029992 | 0.020 | 0.029119 |
| 0.617114 | 0.014603 | 0.023664 | 0.005 | 0.023117 |
| 0.566206 | 0.014197 | 0.025073 | 0.005 | 0.02446 |
| 0.647472 | 0.021047 | 0.032506 | 0.014 | 0.031483 |
| 0.539891 | 0.020598 | 0.038152 | 0.016 | 0.03675 |
| 0.393107 | 0.026934 | 0.068517 | 0.047 | 0.064123 |
| 0.553698 | 0.028588 | 0.051631 | 0.041 | 0.049096 |
| 0.408784 | 0.029433 | 0.072001 | 0.059 | 0.067165 |
| 0.453102 | 0.040658 | 0.089731 | 0.138 | 0.082343 |
| 0.539695 | 0.049349 | 0.091439 | 0.207 | 0.083778 |
| 0.474281 | 0.042088 | 0.088741 | 0.146 | 0.081508 |
| 0.48266 | 0.038069 | 0.078874 | 0.107 | 0.073107 |
| 0.392174 | 0.026541 | 0.067677 | 0.045 | 0.063387 |
| 0.44705 | 0.036886 | 0.082509 | 0.105 | 0.07622 |
| 0.682855 | 0.0141 | 0.020648 | 0.004 | 0.02023 |
| 0.463463 | 0.017698 | 0.038186 | 0.012 | 0.036782 |
| 0.965734 | 0.01692 | 0.017521 | 0.005 | 0.017219 |
| 1.057503 | 0.017434 | 0.016486 | 0.005 | 0.016219 |
| 0.70215 | 0.015236 | 0.021699 | 0.005 | 0.021238 |
| 0.760062 | 0.015973 | 0.021015 | 0.005 | 0.020583 |
| 0.712821 | 0.015311 | 0.02148 | 0.005 | 0.021028 |
| 0.594127 | 0.015895 | 0.026754 | 0.007 | 0.026057 |
| 0.723727 | 0.015388 | 0.021262 | 0.005 | 0.020819 |
| 0.998321 | 0.025553 | 0.025596 | 0.017 | 0.024957 |
| 1.113545 | 0.017734 | 0.015925 | 0.005 | 0.015676 |
| 1.076948 | 0.017539 | 0.016286 | 0.005 | 0.016025 |
| 0.796584 | 0.015881 | 0.019936 | 0.005 | 0.019547 |
| 0.849721 | 0.016222 | 0.019091 | 0.005 | 0.018733 |
| 0.460694 | 0.01327 | 0.028804 | 0.005 | 0.027997 |
| 0.761374 | 0.015647 | 0.020551 | 0.005 | 0.020137 |
| 1.099342 | 0.020188 | 0.018363 | 0.007 | 0.018032 |
| 0.739777 | 0.015611 | 0.021103 | 0.005 | 0.020667 |
| 0.509578 | 0.025189 | 0.04943 | 0.030 | 0.047102 |
| 0.396849 | 0.01819 | 0.045835 | 0.015 | 0.043826 |
| 0.561648 | 0.025416 | 0.045252 | 0.028 | 0.043293 |
| 0.438746 | 0.024316 | 0.055423 | 0.031 | 0.052512 |
| 0.400105 | 0.012397 | 0.030985 | 0.005 | 0.030054 |
| 0.437781 | 0.015628 | 0.035699 | 0.009 | 0.034468 |

**Supplementary Table A. 3.** Classification of samples in hydraulic flow unit 3.

| **FZI** | **RQI** | **φ_z_** | **K** | **φ** |
| --- | --- | --- | --- | --- |
| 1.20843 | 0.068399 | 0.056601 | 0.254 | 0.053569 |
| 1.664173 | 0.027211 | 0.016351 | 0.012 | 0.016088 |
| 1.355081 | 0.048328 | 0.035665 | 0.082 | 0.034436 |
| 1.27402 | 0.035434 | 0.027813 | 0.034 | 0.02706 |
| 1.599828 | 0.07318 | 0.045742 | 0.238 | 0.043741 |
| 1.561474 | 0.019828 | 0.012698 | 0.005 | 0.012539 |
| 1.287851 | 0.018606 | 0.014447 | 0.005 | 0.014241 |
| 1.442964 | 0.019318 | 0.013387 | 0.005 | 0.013211 |
| 1.23215 | 0.018336 | 0.014881 | 0.005 | 0.014663 |
| 1.666327 | 0.055786 | 0.033478 | 0.102 | 0.032394 |
| 1.145359 | 0.017899 | 0.015628 | 0.005 | 0.015387 |
| 1.25768 | 0.01846 | 0.014678 | 0.005 | 0.014466 |
| 1.133955 | 0.01784 | 0.015733 | 0.005 | 0.015489 |
| 1.704415 | 0.020051 | 0.011764 | 0.005 | 0.011627 |
| 1.230088 | 0.029196 | 0.023735 | 0.020 | 0.023185 |
| 1.235458 | 0.035818 | 0.028992 | 0.037 | 0.028175 |
| 1.428609 | 0.025044 | 0.017531 | 0.011 | 0.017229 |
| 1.342811 | 0.018864 | 0.014048 | 0.005 | 0.013854 |
| 1.841193 | 0.020939 | 0.011372 | 0.005 | 0.011244 |
| 1.704486 | 0.021053 | 0.012352 | 0.005 | 0.012201 |
| 1.626425 | 0.020097 | 0.012357 | 0.005 | 0.012206 |
| 1.695125 | 0.029701 | 0.017521 | 0.015 | 0.01722 |
| 1.500615 | 0.030924 | 0.020608 | 0.020 | 0.020192 |
| 1.1204 | 0.01777 | 0.01586 | 0.005 | 0.015612 |
| 1.764982 | 0.020648 | 0.011699 | 0.005 | 0.011563 |
| 1.88211 | 0.03218 | 0.017098 | 0.018 | 0.016811 |
| 1.49191 | 0.019532 | 0.013092 | 0.005 | 0.012923 |
| 1.338218 | 0.018843 | 0.01408 | 0.005 | 0.013885 |
| 1.691242 | 0.020358 | 0.012038 | 0.005 | 0.011894 |
| 1.4544 | 0.036265 | 0.024935 | 0.032 | 0.024328 |

**Supplementary Table A. 4.** Classification of samples in hydraulic flow unit 4.
